# Transforming growth factor beta signaling and craniofacial development: modeling human diseases in zebrafish

**DOI:** 10.3389/fcell.2024.1338070

**Published:** 2024-02-07

**Authors:** Sabrina C. Fox, Andrew J. Waskiewicz

**Affiliations:** Department of Biological Sciences, University of Alberta, Edmonton, AB, Canada

**Keywords:** TGF-β, BMP, signal transduction, craniofacial development, disease, zebrafish

## Abstract

Humans and other jawed vertebrates rely heavily on their craniofacial skeleton for eating, breathing, and communicating. As such, it is vital that the elements of the craniofacial skeleton develop properly during embryogenesis to ensure a high quality of life and evolutionary fitness. Indeed, craniofacial abnormalities, including cleft palate and craniosynostosis, represent some of the most common congenital abnormalities in newborns. Like many other organ systems, the development of the craniofacial skeleton is complex, relying on specification and migration of the neural crest, patterning of the pharyngeal arches, and morphogenesis of each skeletal element into its final form. These processes must be carefully coordinated and integrated. One way this is achieved is through the spatial and temporal deployment of cell signaling pathways. Recent studies conducted using the zebrafish model underscore the importance of the Transforming Growth Factor Beta (TGF-β) and Bone Morphogenetic Protein (BMP) pathways in craniofacial development. Although both pathways contain similar components, each pathway results in unique outcomes on a cellular level. In this review, we will cover studies conducted using zebrafish that show the necessity of these pathways in each stage of craniofacial development, starting with the induction of the neural crest, and ending with the morphogenesis of craniofacial elements. We will also cover human skeletal and craniofacial diseases and malformations caused by mutations in the components of these pathways (e.g., cleft palate, craniosynostosis, etc.) and the potential utility of zebrafish in studying the etiology of these diseases. We will also briefly cover the utility of the zebrafish model in joint development and biology and discuss the role of TGF-β/BMP signaling in these processes and the diseases that result from aberrancies in these pathways, including osteoarthritis and multiple synostoses syndrome. Overall, this review will demonstrate the critical roles of TGF-β/BMP signaling in craniofacial development and show the utility of the zebrafish model in development and disease.

## Introduction

Craniofacial abnormalities, including malformations of the palate, lip, jaw, or cranium (skull), are the most common type of congenital disease, and can severely impact the quality of life of affected individuals by impairing speaking, breathing, eating, or, in some cases, cognition and brain function (Gorlin et al., 2001). The human craniofacial skeleton is generated during embryonic development from a transient population of embryonic cells known as neural crest cells (Jiang et al., 2002; Yoshida et al., 2008). Aberrations to the neural crest, caused by mutations in genes, perturbations to the intrauterine environment, or a combination of both, are known to cause craniofacial abnormalities in infants and children (Gorlin et al., 2001). However, the causative mutations, environmental insults, or interactions between the two remain incompletely understood. Additionally, many of the current treatments for craniofacial abnormalities (such as orofacial clefting and craniosynostosis) involve invasive surgeries with very few preventative measures existing (Shkoukani et al., 2014; Stanton et al., 2022). Therefore, understanding the underlying biology of craniofacial development will aid in understanding the pathogenesis of craniofacial abnormalities, which, in turn, can accelerate the development of strategies for identifying, diagnosing, and preventing craniofacial abnormalities in humans. Vertebrate model organisms, including the mouse and zebrafish, have been vital in understanding craniofacial development, and much of what we know about the underlying pathogenesis of craniofacial abnormalities has come from studies conducted in these organisms. Although many animal models have contributed to our understanding craniofacial development, studies conducted in zebrafish have provided important insights into the genetics and development of craniofacial biology and disease.

Craniofacial development is very complex and requires the coordinated deployment of many distinct cellular processes, including differentiation, migration, adhesion, shape changes, division, and programmed death. These processes must be controlled both spatially and temporally in the embryo to ensure proper development of the craniofacial skeleton; failure of proper temporo-spatial regulation causes abnormal craniofacial development. Signaling pathways are the primary way in which coordination between biological processes is achieved. In particular, the Transforming Growth Factor β (TGF-β) and Bone Morphogenetic Protein (BMP) signaling pathways have well-defined roles in craniofacial development and disease, and their roles in these processes have been illuminated by studies conducted using zebrafish.

In this review, we will examine the utility of using zebrafish as a model organism for understanding the role of BMP and TGF-β signaling in craniofacial development and disease. First, we will describe the components and regulation of TGF-β and BMP signaling pathways. Next, we will present an overview of zebrafish craniofacial development and highlight the advantages of using a zebrafish model for studying craniofacial development. We will then outline craniofacial and skeletal diseases in humans that are frequently caused by defects in BMP/TGF-β signaling. Finally, we will review the zebrafish studies of BMP and TGF-β signaling in craniofacial development and their impact on understanding the pathogenesis of craniofacial and skeletal disease.

## Transforming growth factor Beta signaling

### Ligands

TGF-β was first identified in the late 1970s/early 1980s as one of two factors (the other being TGF-α) that were able to “transform” anchorage-dependent fibroblasts into morphologically distinct cells able to grow in soft agar (De Larco and Todaro, 1978; Moses et al., 1981; Roberts et al., 1981). Shortly after the cDNAs for TGF-β1, 2, and 3 were cloned, several other proteins that shared sequence similarity with the C-terminal sequences of TGF-βs were identified (Derynck et al., 1985; Derynck et al., 1988; Mason et al., 1985; Cate et al., 1986; de Martin et al., 1987; Marquardt et al., 1987; Padgett et al., 1987; Weeks and Melton, 1987; Wozney et al., 1988). Currently, the TGF-β superfamily of signaling molecules contains at least 33 members in mammals, making it one of the largest families of signaling proteins. On the basis of structure and signaling activity, the TGF-β superfamily can be subdivided into a series of subfamilies. Six ligands comprise the TGF-β/Lefty/Inhibin family including the canonical TGF-βs (TGF-β1, 2, and 3), Lefty A and B, and Inhibin alpha (Hinck, 2012; Hinck et al., 2016). Activins comprise four additional ligands (Actβa, Actβb, Actβc, Actβe) (Hinck, 2012; Hinck et al., 2016). There are 22 members of the BMP/Growth Differentiation Factor (GDF)/Nodal/Mullerian Inhibiting Substance (MIS) family, which can be organized into four subfamilies based on structure (Newfeld et al., 1999; Hinck, 2012; Hinck et al., 2016). Eight ligands (BMP2, 4, 5, 6, 7, 8, and GDF1 and 3) comprise the canonical BMPs (Newfeld et al., 1999; Hinck, 2012; Hinck et al., 2016). A separable subfamily consists of GDF5, 6, and 7 together with BMP9 and 10. The three ligands Nodal, BMP3 and BMP10 (GDF2) are also structurally similar and represent a distinct subfamily of BMPs (Newfeld et al., 1999; Hinck, 2012; Hinck et al., 2016). More divergent from the above are the loosely grouped ligands BMP15, GDF15, GDF9, and MIS (Newfeld et al., 1999; Hinck, 2012; Hinck et al., 2016).

**FIGURE 1:**
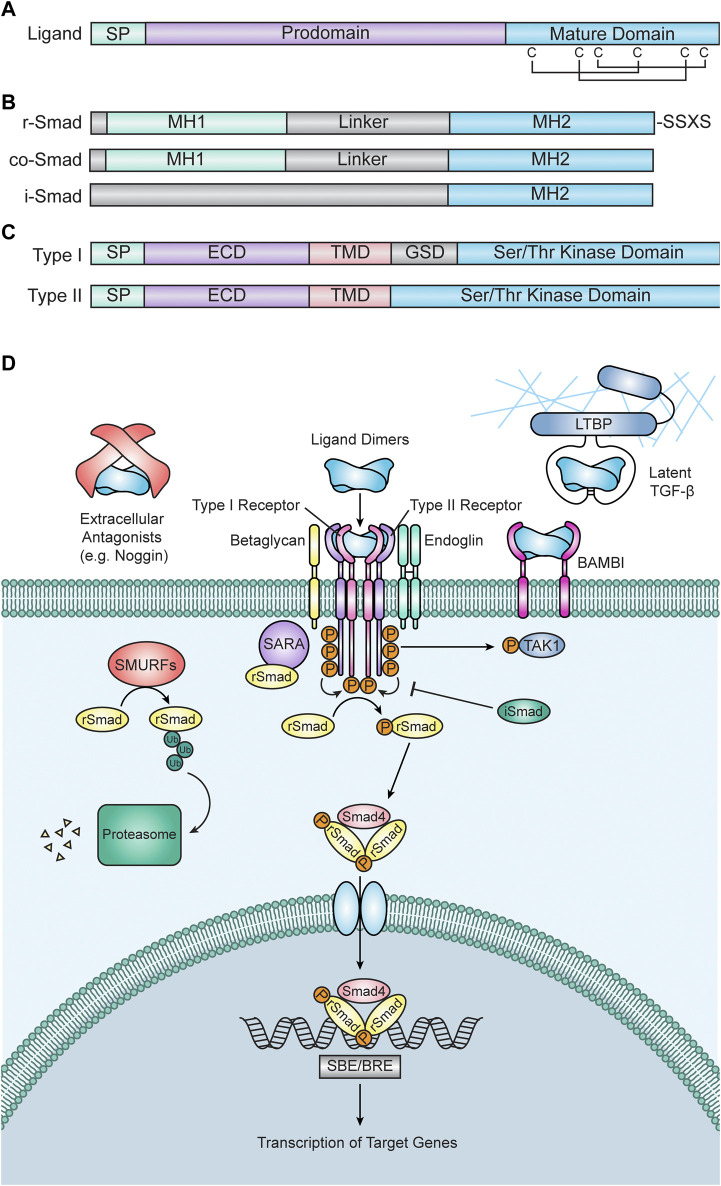
Transforming Growth Factor Beta (TGF-β) Superfamily Signaling. **(A)** Diagram of a prototypical TGF-β superfamily ligand. Each ligand consists of a short signal peptide (SP), followed by a prodomain and the mature signaling domain. The mature domain is stabilized by a “cysteine knot” motif, which consists of six cysteine residues connected via disulfide bonds. **(B)** Diagrams of Smad proteins. Receptor associated Smads (r-Smads, top) have an N-terminal Mad Homology 1 (MH1) domain and a C-terminal Mad Homology 2 (MH2) domain separated by a linker domain. R-Smads also contain an SXSS motif at their C-terminus, which is phosphorylated by receptors to initiate intracellular signal transduction. Common Smad4 (co-Smad, middle) has a similar structure to r-Smads but is lacking a C-terminal SXSS motif. Inhibitory Smads (i-Smads, bottom) contain an MH2 domain but lack an MH1 domain, allowing them to interact with r-Smad binding partners but preventing them from binding DNA and activating gene expression. **(C)** Diagrams of receptors. Type I receptors (top) contain a signal peptide (SP), an extracellular domain (ECD), a transmembrane domain (TMD), a glycine/serine-rich domain (GSD), and a serine/threonine kinase domain. Type II receptors (bottom) have a similar structure to Type I receptors, but they lack a GSD. The protein structures in **(A–C)** are highly conserved among vertebrates, including zebrafish. **(D)** TGF-β signal transduction. Signal transduction is initiated when TGF-β ligand dimers bind to two type II and two type I receptors. Ligand-receptor binding can be inhibited by extracellular antagonists (e.g., Noggin), TGF-β protein latency, or the BMP and Activin Membrane Bound Inhibitor (BAMBI). Ligand-receptor binding is also frequently facilitated by the coreceptors Endoglin or Betaglycan. Once the ligand-receptor complex is formed, constitutively active type II receptors phosphorylate type I receptors, thus activating them. Active type I receptors then phosphorylate r-Smads, which allow them to interact with co-Smad4 to create a trimeric Smad complex. R-Smad phosphorylation is facilitated by Smad Anchor for Receptor Activation (SARA) and inhibited by i-Smads. Type I receptors can also phosphorylate non-Smad targets, including TGF-β Activated Kinase (TAK1). Additionally, the degradation of r-Smads is promoted by Smad Ubiquitination Regulatory Factors (SMURFs). Once formed, Smad trimers enter the nucleus, bind to Smad Binding Elements (SBEs) or BMP Response Elements (BREs), and regulate the transcription of target genes.

As alluded to previously, TGF-β superfamily ligands share a prototypical structure that is vital to their signaling activity and function. All ligands contain an N-terminal signal peptide, followed by a large (∼250 amino acid) prodomain that is necessary for protein folding, processing, and, in some cases, protein regulation and a smaller (∼110 amino acid) C-terminal mature growth factor domain (Figure 1A). TGF-β ligands form a structure known as a “cysteine knot” in their mature growth factor domain, where four distal polypeptide sequences are linked together by three disulfide bonds between six closely spaced pairs of cysteines (McDonald and Hendrickson, 1993) (Figure 1A). Ligand monomers are also covalently bound via their C-terminal signaling domains by an additional disulfide linkage, resulting in a ligand dimer with a structure that is frequently likened to a “butterfly” or two “hands” (Gentry et al., 1988). This “hand” structure consists of two sets of β-sheets that form “fingers” attached to a central stabilizing “wrist” or “palm” composed of an α-helix (Hinck et al., 2016). After transit out of the ER and into the Golgi, the prodomain is cleaved from the mature domain (Gentry et al., 1988). The prodomain typically remains associated with the mature signaling ligand and there is significant evidence that prodomains are critical for regulation of signaling activity in many instances, as suggested by 3D models of prodomain-mature ligand complexes (Gray and Mason, 1990).

### Receptors

TGF-β signaling is initiated by binding of a ligand to its cognate type I and type II receptors. Receptors have two domains: an extracellular ligand-binding ectodomain and an intracellular kinase domain (Figure 1B). Unlike other receptor kinases, which typically phosphorylate tyrosine (e.g., fibroblast growth factor (FGF) receptors), TGF-β receptors are serine/threonine kinases (Wozney et al., 1988; Georgi et al., 1990; Mathews and Vale, 1991; Lin et al., 1992; Baarends et al., 1994; Kawabata et al., 1995; Yoshida et al., 2008). Receptors are categorized by which class of ligand they bind (Summarized in Table 1) (Hinck, 2012). Binding of a TGF-β ligand to its cognate type I and II receptors results in the creation of a receptor-ligand complex wherein a ligand dimer, via its receptor-binding domain, interacts with two type II receptors and two type I receptors (Wrana et al., 1992; Yamashita et al., 1994). This complex brings the type I and II receptors into proximity of one another, allowing the constitutively active serine/threonine kinase domain of the type II receptors to phosphorylate a glycine- and serine-rich domain (GS domain) in the cytoplasmic tail of the type I receptors, thus activating them and allowing them to phosphorylate downstream targets (Wrana et al., 1992; Wrana et al., 1994) (Figure 1D).

**TABLE 1:** Summary of TGF-β/BMP signaling pathway components. Listed below are each subgroup of ligands, followed by their cognate type I and type II receptors and the Smad proteins that they activate. Table adapted from information provided by Hinck (2012).

Ligands	Type I receptors	Type II receptors	Smads
TGF-β1-3	TGFBR1	TGFBR2	Smad2/Smad3
Activinβa/b/c/e	TGFBR1, ACVR1B	ACVR2A, ACVR2B	Smad2/Smad3
Nodal	ACVR1B, ACVR1C	ACVR2A, ACVR2B	Smad2/Smad3
MIS (AMH)	BMPR1A, BMPR1B, ACVR1C	AMHR2	Smad1/5/8
BMPs	BMPR1A, BMPR1B, ACVRL1, ACVR1	BMPR2, ACVR2A, ACVR2B	Smad1/5/8
GDFs	BMPR1A, BMPR1B, ACVRL1, ACVR1	BMPR2, ACVR2A, ACVR2B	Smad1/5/8

### Smads

Once activated, type I receptors phosphorylate their intracellular target proteins, the receptor-associated Smads (r-Smads). Vertebrate Smads are homologous to (and named after) the Sma and Mad proteins, identified as regulators of TGF-β signaling in *C. elegans* and *D. melanogaster,* respectively (Raftery et al., 1995; Sekelsky et al., 1995; Derynck et al., 1996; Savage et al., 1996) Vertebrates possess five distinct r-Smads: Smad1, Smad2, Smad3, Smad5, and Smad8/9. Smad2/3 are typically activated by TGF-β, Activin, and Nodal ligands and receptors, whereas Smad1/5/8/9 are typically activated by BMP/GDF ligands and receptors (Table 1) (Graff et al., 1996). r-Smads have C-terminal and N-terminal Mad Homology (MH) domains, termed MH1 and MH2 respectively, which are connected by a linker domain that serves as a hub for facilitating cross talk between other pathways and regulating Smad protein activity (Fuentealba et al., 2007; Sapkota et al., 2007; Macias et al., 2015) (Figure 1C). Prior to phosphorylation, Smad2 interacts with the protein SARA (Smad Anchor for Receptor Activation), which anchors Smad2 to the cell membrane and facilitates the interaction between Smads and active Type I receptors (Tsukazaki et al., 1998) (Figure 1D). Similarly, the endosome-associated protein Endofin binds to Smad1 and enhances its phosphorylation by BMP receptors (Shi et al., 2007).

Active type I receptors interact with Smads via their MH1 domain and phosphorylate an SSXS motif on their C-terminus (Macías-Silva et al., 1996; Abdollah et al., 1997; Kretzschmar et al., 1997; Souchelnytskyi et al., 1997) (Figure 1C). This phosphorylation allows r-Smads to form a trimeric complex with common Smad4 (co-Smad4) consisting of two r-Smad subunits and one Smad4 subunit (Lagna et al., 1996; Kretzschmar et al., 1997; Wu et al., 1997; Zhang et al., 1997; Kawabata et al., 1998) (Figure 1D). Once formed, this Smad complex enters the nucleus and binds DNA elements to facilitate the transcription of target genes (Baker and Harland, 1996; Hoodless et al., 1996; Liu et al., 1996). Generally, Smad3/Smad4 complexes bind AGAC or GTCT sequences (termed Smad Binding Sequences or SBEs), whereas Smad1/Smad4 complexes bind SBEs weakly and preferentially bind GC-rich sequences (termed BMP Response Elements or BREs) (Zawel et al., 1998; Katagiri et al., 2002).

In addition to the r-Smads and co-Smad4, two additional Smad proteins exist: Smad6 and Smad7, also known as inhibitory Smads (i-Smads). As their name suggests, these Smad proteins inhibit signal transduction (Hayashi et al., 1997; Imamura et al., 1997; Nakao et al., 1997; Tsuneizumi et al., 1997; Hata et al., 1998) (Figure 1D). Smad6 is a potent inhibitor of BMP signaling, while Smad7 inhibits both TGF-β and BMP signaling (Hayashi et al., 1997; Hata et al., 1998). Although the MH2 domain of i-Smads is similar to other Smads, i-Smads differ significantly in their N-terminal MH1 and linker domains (Hanyu et al., 2001; Macias et al., 2015) (Figure 1C). i-Smads compete with r-Smads for type I receptor occupancy, thus preventing r-Smads from being phosphorylated and therefore inhibiting signal transduction (Nakao et al., 1997; Goto et al., 2007). Additionally, Smad7 can recruit other inhibitory proteins to type I receptors, including E3 ubiquitin ligases such as SMURFs, further attenuating signaling (Kavsak et al., 2000; Ebisawa et al., 2001). There is also evidence that i-Smads inhibit Smad-dependent transcription, suggesting that i-Smads interfere with signal transduction at multiple levels (Yan et al., 2016). The expression of i-Smads is induced by the pathways that they inhibit, thereby generating a negative feedback loop (Nagarajan et al., 1999; Denissova et al., 2000; Ishida et al., 2000; Benchabane and Wrana, 2003).

Distinct from the canonical Smad pathway, there is significant evidence that the TGF-β superfamily transduces its signal through non-Smad pathways. BMP/TGF-β signaling have been shown to induce the phosphorylation of mitogen activated protein kinases (MAPKs), including TGF-β Activated Kinase 1 (TAK1), which have been shown to activate kinases with downstream effects ranging from transcriptional regulation to cytoskeletal rearrangements (Yamaguchi et al., 1995) (Figure 1D). Additionally, several components of the TGF-β pathway have been shown to crosstalk with other signaling pathways, further adding complexity to these pathways and signaling effectors (Reviewed by Luo, 2017).

### Regulators of TGF-β signaling

#### TGF-β proprotein latency

Unlike other superfamily members, TGF-β1, 2, and 3 are synthesized as latent protein complexes which only become activated after an activation cascade (Pircher et al., 1984; Lawrence et al., 1985; Wakefield et al., 1988; 1989; Lyons et al., 1990). After cleavage by proteases in the Golgi, TGF-β1-3 remain noncovalently associated with their prodomains (Gentry and Nash, 1990). In this context, the prodomain confers latency to the ligand and is referred to as Latency Associated Peptide (LAP). LAP masks the amino acids on the mature ligand that are critical for receptor binding, thus inhibiting interactions between the ligand and receptors (Shi et al., 2011). During synthesis and secretion, LAP becomes covalently linked to Latent TGF-β Binding Proteins (LTBP), which typically associate closely with components of the ECM such as fibrillin and fibronectin (Kanzaki et al., 1990) (Figure 1D). Secreted matrix metalloproteinases and plasmin degrade both the LAP and corresponding LTBP, thereby allowing the ligand to bind its cognate receptor (Sato and Rifkin, 1989; Yu and Stamenkovic, 2000). In addition, LAPs contain an Arg-Gly-Lys (RGD) motif, which is recognized by membrane-bound integrins. Upon binding of integrin dimers to LAPs, the mechanical force generated by the actin cytoskeleton induces a conformational change that releases the mature dimer from its LAP (Munger et al., 1999; Annes et al., 2004).

#### Extracellular BMP antagonists

While proprotein latency is unique to the TGF-β subfamily, inhibition of receptor binding by extracellular antagonists is unique to the BMP subfamily. BMP antagonists consist of subfamilies of secreted proteins and include Noggin, Chordin, Gremlin, DAN, Cerberus, and Follistatin (Zimmerman et al., 1996; Piccolo et al., 1999). Secreted BMP antagonists modulate binding of BMP dimers to their receptors (Groppe et al., 2002; Harrington et al., 2006; Zhang et al., 2008; Nolan et al., 2013). Like LAPs, BMP antagonists bind to BMP ligands and mask the residues that are critical for receptor binding, thus preventing binding of the ligand to its receptors (Groppe et al., 2002; Harrington et al., 2006; Zhang et al., 2008; Nolan et al., 2013) (Figure 1D). BMP antagonists tend to be expressed in very restricted temporal and spatial patterns, and the expression of antagonists is often regulated by other signaling pathways. Therefore, BMP antagonists are significant mediators of signaling crosstalk.

#### Co-receptors

In addition to the canonical type I and type II receptors that directly mediate signal transduction, there are also several co-receptors that modulate receptor activity. Some well-studied examples of these co-receptors are betaglycan and endoglin (Figure 1D). Betaglycan (sometimes referred to as the Type III TGF-β receptor or TGFBRIII) is a membrane-bound glycoprotein that binds TGF-β ligands, which can have a low affinity for their cognate type I and II receptors, and brings them into proximity of their receptors to form an active signaling complex (Wang et al., 1991; López-Casillas et al., 1993). Alternatively, the ectodomain of betaglycan can be cleaved and act as a sink for ligands, thereby preventing them from binding to receptors in a manner similar to extracellular BMP antagonists or LAP (López-Casillas et al., 1994). Endoglin, whose expression is primarily restricted to endothelial cells, is another well studied co-receptor for TGF-β (Cheifetz et al., 1992; Yamashita et al., 1994). Endoglin’s role in modulating signal transduction is complex; it can either antagonize signaling or promote ligand binding (Lastres et al., 1996). Like betaglycan, the ectodomain of endoglin can be cleaved and potentially regulate the availability of ligands in the extracellular space (Li et al., 1998; Castonguay et al., 2011). More recently, a BMP-specific co-receptor, Neogenin, was discovered; Neogenin binds BMP2, 4, 6, and 7, and enhances the ability of these ligands to activate both Smad1/5/8 phosphorylation and RhoA activity (Hagihara et al., 2011). Additionally, other membrane-bound or membrane-associated regulators of receptor activity exist, including GPI-anchored proteins such as Cripto, and have been shown to regulate TGF-β superfamily signaling in a cell-type specific manner (Minchiotti et al., 2000; Yeo and Whitman, 2001; Cheng et al., 2003; Gray et al., 2003; Garcia de Vinuesa et al., 2021).

#### BAMBI

Perhaps one of the most well-studied and potent negative regulators of TGF-β signaling is by BMP and Activin Membrane Bound Inhibitor (BAMBI). BAMBI expression is activated by TGF-β signaling, thereby creating a feedback loop where TGF-β signaling inhibits itself (Karaulanov et al., 2004; Sekiya et al., 2004). Structurally, BAMBI is very similar to TGF-β receptors, but lacks a GS domain in its cytoplasmic tail, thereby preventing it from being phosphorylated by Type II receptors and blocking signaling (Onichtchouk et al., 1999). BAMBI interacts with and acts as a sink for receptors, thereby preventing them from forming an active receptor complex (Onichtchouk et al., 1999) (Figure 1D). BAMBI also recruits i-Smads to the cell membrane, allowing them to interact with active receptors and inhibit signaling (Yan et al., 2009) (Figure 1D).

#### Regulation of Smads

Two main mechanisms exist to downregulate signaling at the level of Smads: dephosphorylation of activated r-Smads and proteasomal degradation of r-Smads. Once in their phosphorylated state, r-Smads can be dephosphorylated by phosphatases, which leads to the deactivation of the active Smad complex and, therefore, attenuation of signaling (Bruce and Sapkota, 2012). The second mechanism by which r-Smad activity is regulated is by degradation in the proteasome. Like other intracellular proteins, Smads can be covalently bound to ubiquitin by E3 ubiquitin ligases and targeted to the proteasome for degradation. Smad-specific E3 ubiquitin ligases, including SMURF1 and SMURF2, add ubiquitin groups to Smads, thereby targeting them for degradation (Zhu et al., 1999; Lin et al., 2000; Zhang et al., 2001) (Figure 1D). Although ubiquitination and phosphorylation represent the most extensively studied post-translational modifications of Smads, there is evidence that sumoylation, acetylation, and poly-ADP-ribosylation all play a role in regulating Smad activity.

### Zebrafish TGF-β signaling

Although TGF-β/BMP pathways show a high degree of conservation between mammals and zebrafish, there are a few notable differences between the zebrafish and mammalian pathways. For example, the zebrafish homolog of BMP2 (Bmp2b) appears to be the functional equivalent to BMP4 in other systems (Kishimoto et al., 1997). Additionally, teleost fishes, including zebrafish, have undergone a complete genome duplication event, resulting in many genes (including those encoding components of the TGF-β/BMP pathways) having 2 paralogs (e.g., *bmp2a* and *bmp2b*, *gdf6a*, and *gdf6b*, etc.) (Glasauer and Neuhauss, 2014). In some instances, one paralog takes over the ancestral function, while the other undergoes non-functionalization (Glasauer and Neuhauss, 2014). For example, *gdf6a* appears to perform the same function as *GDF6/Gdf6,* whereas *gdf6b* appears to be, for all intents and purposes, non-functional (Glasauer and Neuhauss, 2014; Gramann et al., 2019). In other instances, the ancestral functions of the paralogs sub-functionalize, wherein each paralog performs regulates a slightly different process (Glasauer and Neuhauss, 2014). For instance, zebrafish have three paralogs of Noggin (*nog1/2/3*), each with completely different expression patterns and (presumably) different functions (Fürthauer et al., 1999).

## Zebrafish craniofacial development

### The cranial neural crest

**FIGURE 2:**
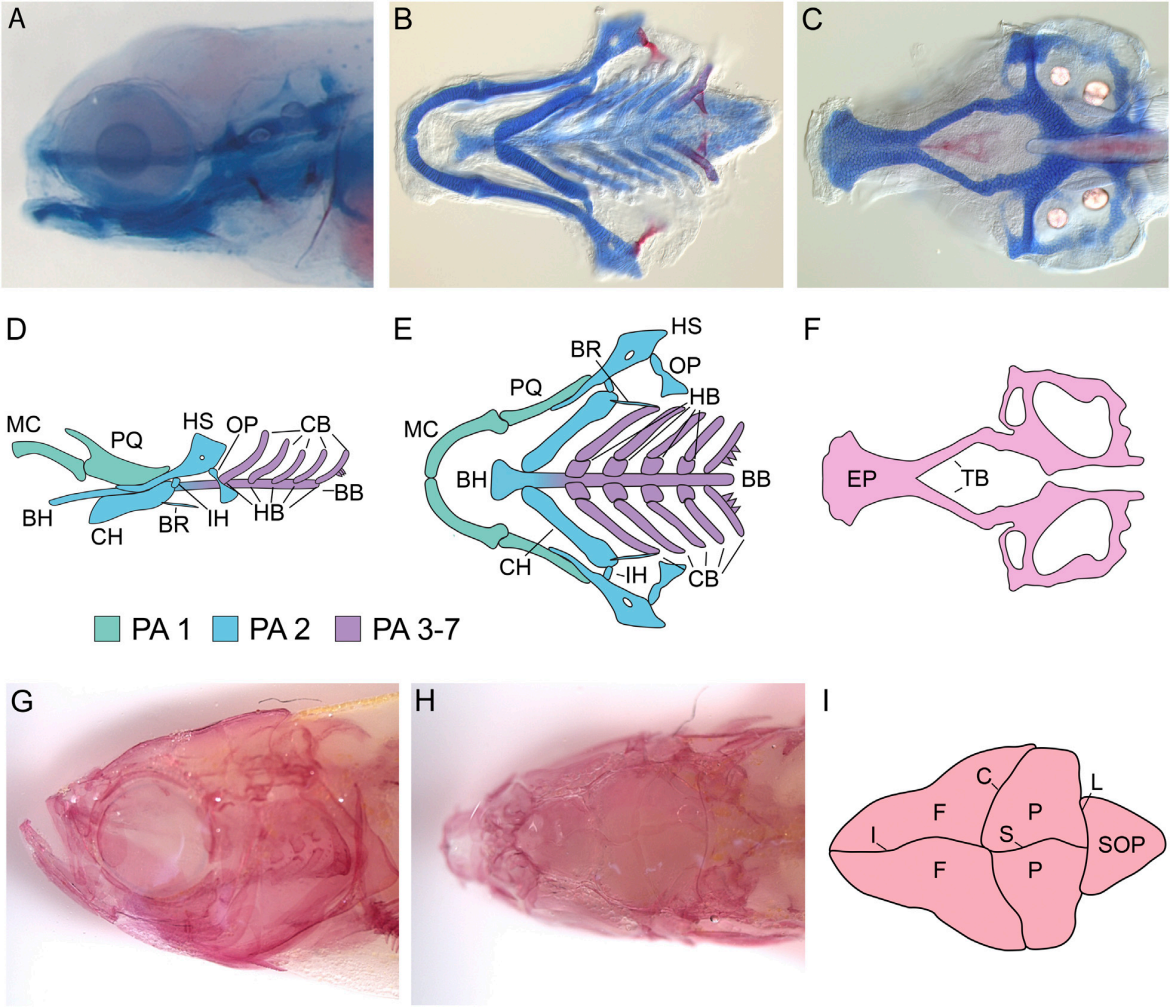
Zebrafish Craniofacial Anatomy. **(A)** Stereomicroscope image of a 5 dpf zebrafish larvae stained with Alcian blue (cartilage) and Alizarin red (mineralized bone). All cartilage elements and many of the early bone elements are present by 5 dpf in zebrafish, making analysis of craniofacial phenotypes relatively easy. **(B,C)** DIC microscopy image of the dissected, flat-mounted viscerocranium **(B)** and neurocranium **(C)** from a 5 dpf larval zebrafish stained with Alcian blue and Alizrin red. Flat-mounts of the larval craniofacial skeleton reveal the structural properties of cartilage/bone that are not visible by stereomicroscopy. **(D,E)** Schematics of the zebrafish viscerocranium at 5dpf. Schematics are shown in both a lateral **(D)** and ventral **(E)** orientation. At 5 dpf, the following cartilage structures are present in the viscerocranium: Meckel’s cartilage (MC) and the palatoquadrate (PQ) are derived from first pharyngeal arch (PA) neural crest, the basihyal (BH), ceratohyal (CH), hysoymplectic (HS), and the interhyal (IH) are derived from second PA neural crest, and the hypobranchials (HB), ceratobranchials (CB), and basibranchials (BB) are derived from third to seventh PA neural crest. At this stage, several mineralized membrane bones are also present, including the opercle (OP) and the branchiostegal ray (BR). **(F)** Schematic of the zebrafish neurocranium at 5dpf. At 5 dpf, both the ethmoid plate (EP) and the trabeculae (TB) are visible and the posterior part of the neurocranium is beginning to form. **(G–I)** Adult zebrafish craniofacial anatomy. **(G)** Lateral view of the adult zebrafish craniofacial skeleton stained with Alizarin red and visualized by stereomicroscopy. By adulthood, the larval craniofacial skeleton has been elaborated on significantly. **(H)** Dorsal view of the adult zebrafish craniofacial skeleton visualized by stereomicroscopy. Dorsal views reveal the zebrafish calvaria (skull bones) and sutures. **(I)** Schematic of adult zebrafish calvaria and sutures. Adult zebrafish possess two frontal bones (F), two parietal bones (P), and a supraoccipital bone (SOP) separated by the interfrontal suture (IF), the coronal suture (C), the sagittal suture (S), and the lamboid suture (L). All images are oriented with anterior facing left.

The vertebrate head skeleton is made up of two components: the neurocranium, which houses the brain and sensory organs, and the viscerocranium, which includes the midface, jaw, and posterior pharyngeal structures (Schilling and Kimmel, 1994). Like other vertebrates, much of the zebrafish craniofacial skeleton is derived from a subpopulation of neural crest cells termed the cranial neural crest. During gastrulation, a region of ectoderm immediately lateral to the neural plate (the presumptive nervous system), termed the neural plate border, is specified by inductive signals that pattern the dorsal-ventral axis of the zebrafish embryo (Woo and Fraser, 1995). These inductive signals will activate the expression of neural plate border specifier genes, which predominantly encode transcription factors (e.g., *pax3a, tfap2a, zic3, dlx5/6, msx1a/1b/3,* etc.) (Seo et al., 1998; Knight et al., 2003; Knight et al., 2004; Barrallo-Gimeno et al., 2004; Phillips et al., 2006; Garnett et al., 2012; Narboux-Neme et al., 2019). NPB specifiers, in turn, will drive the expression of additional transcription factors that are required for NC identity (e.g., *foxd3, snail1b, soz10, sox9a/b, twist,* etc.) (Thisse et al., 1995; Kelsh and Eisen, 2000; Dutton et al., 2001; Li et al., 2002; Yan et al., 2002; 2005; Suazo et al., 2004; Carney et al., 2006; Lister et al., 2006; Montero-Balaguer et al., 2006; Stewart et al., 2006; Germanguz et al., 2007; Gestri et al., 2009; Das and Crump, 2012). During neurulation, neural crest cells will undergo an epithelial to mesenchymal transition (EMT), in which they detach from the ectoderm and initiate migration. Like other vertebrates, zebrafish EMT is a complex process that involves changes in the morphological and adhesive properties of neural crest cells; changes in these properties are primarily attained from upregulation of EMT genes by neural crest specifier genes (Berndt et al., 2008; Clay and Halloran, 2013; Clay and Halloran, 2014; Jimenez et al., 2016; Ahsan et al., 2019). After EMT, neural crest cells will migrate through the head mesenchyme and form the zebrafish craniofacial skeleton. A subpopulation of neural crest cells from the midbrain will migrate around the eyes and populate the frontonasal and maxillary prominences, where they will give rise to the ethmoid plate, trabeculae, and anterior skull vault of the neurocranium (Wada et al., 2005; Dougherty et al., 2013). Neural crest from the midbrain and rhombomeres of the hindbrain will migrate more ventrally and infiltrate the pharyngeal arches (PAs) (Wada et al., 2005; Dougherty et al., 2013). Neural crest cells from the midbrain and rhombomeres 1–3 populate PA1 (the mandibular arch), neural crest cells from rhombomeres 3–5 populate PA2 (the hyoid arch), and neural crest cells from rhombomeres 5–7 populate PA3-7 (the brachial arches) (Schilling and Kimmel, 1994). After migration, neural crest cells will condense and differentiate into cartilage and bone and ultimately form the zebrafish craniofacial skeleton (Schilling and Kimmel, 1994). During the larval stages, the craniofacial skeleton is relatively simple and primarily composed of cartilage with a few intramembranous bones (Cubbage and Mabee, 1996) (Figure 2A). Neural crest cells (and the subsequent cartilage and bone elements that they form) receive inductive signals along the anterior-posterior, dorsal-ventral, and proximal-distal axes, allowing each element to acquire a distinct shape that is dependent on their location (Minoux and Rijli, 2010; Medeiros and Crump, 2012). This, in turn, dictates the function of each element (Minoux and Rijli, 2010; Medeiros and Crump, 2012).

### The zebrafish viscerocranium

Perhaps the best characterized component of the zebrafish craniofacial skeleton is the viscerocranium, which arises from the PAs (Figures 2A, B, D, E). Neural crest cells in the mandibular arch (PA1) give rise to Meckel’s cartilage, a pair of ventral cartilage rods that articulate at the midline via the mandibular symphysis. In larval zebrafish, Meckel’s cartilage acts as the lower jaw, and during adult craniofacial development, it acts as a template for the bones of the adult jaw to condense on (Cubbage and Mabee, 1996; Piotrowski et al., 1996; Schilling et al., 1996; Schilling and Kimmel, 1997). Located more dorsally in the mandibular arch is the palatoquadrate, a fan-shaped cartilage that articulates with Meckel’s cartilage via the mandibular jaw joint (Cubbage and Mabee, 1996; Piotrowski et al., 1996; Schilling et al., 1996; Schilling and Kimmel, 1997). The palatoquadrate is also attached to the ethmoid plate via the anteriorly positioned pterygoid processes to form the larval upper jaw (Cubbage and Mabee, 1996; Piotrowski et al., 1996; Schilling et al., 1996; Schilling and Kimmel, 1997). In the hyoid arch (PA2), ventral neural crest cells will form a pair of rod-shaped cartilage elements called the ceratohyals (Cubbage and Mabee, 1996; Piotrowski et al., 1996; Schilling et al., 1996; Schilling and Kimmel, 1997). Each ceratohyal is connected to the basihyal, an unpaired cartilage element positioned along the ventral midline (Cubbage and Mabee, 1996; Piotrowski et al., 1996; Schilling et al., 1996; Schilling and Kimmel, 1997). The ceratohyal serves to support the larval lower jaw and will progressively ossify via intramembranous ossification throughout larval development (Cubbage and Mabee, 1996; Piotrowski et al., 1996; Schilling et al., 1996; Schilling and Kimmel, 1997). The hyosymplectic is oriented more dorsally in the hyoid arch and articulates with the otic cartilage to connect skeletal elements from the hyoid arch to the cranium (Cubbage and Mabee, 1996; Piotrowski et al., 1996; Schilling et al., 1996; Schilling and Kimmel, 1997). The ceratohyal and hyosymplectic are joined to one another by the interhyal cartilage in the hyoid joint (Cubbage and Mabee, 1996; Piotrowski et al., 1996; Schilling et al., 1996; Schilling and Kimmel, 1997). The brachial arches (PA3-7) give rise to the ceratobranchials, which are paired, rod-shaped cartilages that are connected to the midline basibranchials via small cartilage elements called hypobrachials (Cubbage and Mabee, 1996; Piotrowski et al., 1996; Schilling et al., 1996; Schilling and Kimmel, 1997). These cartilage elements serve to support the gills and associated structures. The craniofacial skeleton undergoes many changes as the zebrafish proceeds through metamorphosis and into adulthood. New membrane bones are formed, and existing ones grow significantly (Cubbage and Mabee, 1996). Several of the cartilage elements ossify via endochondral ossification or are degraded after membrane bones form in their place (Cubbage and Mabee, 1996). The adult zebrafish craniofacial skeleton has 43 bones (compared to ∼22 in mammals), which represents a complex arrangement of highly specialized skeletal structures (Cubbage and Mabee, 1996) (Figure 2G).

### The zebrafish neurocranium

The neurocranium, composed of the ethmoid plate, trabeculae, and the skull vault, also begins development in the embryonic and larval stages. The ethmoid plate forms from bilateral populations of neural crest cells that converge on the midline to produce a flat, fan-shaped sheet of cells (the ethmoid plate) that is connected to the neurocranium via two paired rods of cartilage termed the trabeculae (Wada et al., 2005; Eberhart et al., 2006; Dougherty et al., 2013) (Figures 2C, F). The ethmoid plate articulates with the retroarticular processes of the palatoquadrate to form the larval upper jaw (Cubbage and Mabee, 1996). The ethmoid also serves as the “roof” of the mouth and separates the larval mouth from the rest of the anterior head (Cubbage and Mabee, 1996). Later in development (∼2 months post-fertilization) the zebrafish skull vault develops (Topczewska et al., 2016; Kanther et al., 2019). Zebrafish, like mammals, have 5 calvaria: a pair of frontal bones, a pair of parietal bones, and one supraoccipital bone (Topczewska et al., 2016) (Figures 2H, I). The frontal and parietal bones develop from membranous ossification where mesenchymal cells ossify directly, whereas the supraoccipital bone must first pass through a cartilage template before ossifying via endochondral ossification (Topczewska et al., 2016; Kanther et al., 2019). Like mammals, the anterior-most region of the skull vault is derived from neural crest, whereas the more posterior regions are derived from mesoderm (Kague et al., 2012; Mongera et al., 2013). However, in contrast to mammals, where the demarcation between neural crest and mesoderm is represented by the coronal suture, zebrafish have a cryptic boundary between neural crest-derived and mesoderm-derived bones that occurs somewhere in the middle of the two frontal bones, making the frontal bones dual origin (Kague et al., 2012; Mongera et al., 2013). Calvaria are separated by fibrous joints called sutures (Topczewska et al., 2016; Kanther et al., 2019). The interfrontal suture separates the two frontal bones, the coronal suture separates the frontal and parietal bones, the sagittal suture separates the two parietal bones, and the lamboid sutures separate the parietal bones from the supraoccipital bone (Topczewska et al., 2016) (Figures 2H, I). Sutures contain osteoprogenitor cells, which act as a leading edge for calvaria osteogenesis as the brain grows, and mesenchymal stem cells, which are derived from both the mesoderm and neural crest and provide a niche for calvaria osteogenesis (Topczewska et al., 2016; Kanther et al., 2019). Complex signaling networks and cell behaviors are necessary for patterning suture formation and maintaining patency of the sutures (Topczewska et al., 2016; Kanther et al., 2019). Unlike human sutures, which fuse completely by adulthood, zebrafish sutures remain patent throughout life, allowing for lifelong brain growth (Topczewska et al., 2016).

### Evolutionary conservation of the zebrafish craniofacial skeleton

A significant advantage of using zebrafish as a model to study craniofacial development and disease is the homology between several zebrafish and mammalian craniofacial structures. For instance, many elements in the larval/juvenile craniofacial skeleton, such as Meckel’s cartilage or the cranial sutures, are shared between the zebrafish and its mammalian, avian, and amphibian counterparts. Moreover, even when zebrafish do not have an obvious structure that is present in other animals, they frequently have structures that share an evolutionary origin with structures in mammals and are therefore considered homologous. Two examples of this are the ossicles of the middle ear and the mammalian hard palate. While zebrafish do not produce ossicles, the bones in the inner ear necessary for the conduction of sound, mammalian ossicles evolved from components of the teleost viscerocranium, and these components are homologous to mammalian ossicles (Anthwal et al., 2013). In zebrafish, the hyomandibula is homologous to the mammalian stapes, whereas the posterior of Meckel’s cartilage and the ventral portion of the palatoquadrate are homologous to the malleus and incus (Reichert, 1837). Furthermore, the connection of the hyomandibula to the otic cartilage in fish resembles the connection of the stapes to the oval window in mammals, further suggesting homology between these two structures (Reichert, 1837). The ethmoid plate has also been shown to be homologous to the mammalian hard palate. The ethmoid plate and the mammalian palate both have similar morphogenetic origins; like the mammalian palatal shelves, the neural crest cells that give rise to the ethmoid plate migrate towards the midline and fuse (Wada et al., 2005; Eberhart et al., 2006; Dougherty et al., 2013). Additionally, the genetic architecture that regulates palatogenesis in mammals has been shown to regulate ethmoid plate formation in zebrafish (Swartz et al., 2011). Therefore, these zebrafish structures can be used to model human craniofacial development and disease.

## Zebrafish studies of TGF-β signaling and craniofacial development

As stated previously, the zebrafish model has been an invaluable resource for studying the role of signaling molecules in craniofacial development and disease and complement data generated in mouse models (for an excellent review on the use of mouse models to study craniofacial development and disease, we direct the reader to Ueharu and Mishina, 2023). In this section, we review studies conducted in zebrafish regarding BMP/TGF-β signaling in this process. The major details covered in this section are summarized in Table 2.

**TABLE 2:** Summary of Zebrafish Studies of TGF-β Signaling in Craniofacial Development. Listed below are the key findings generated in zebrafish regarding TGF-β superfamily signaling in craniofacial development organized by developmental processes with their corresponding references. Refs. = References.

Process	BMP/TGF-β involvement	Refs.
Dorsal/Ventral Patterning	Ventral BMP and dorsal *chordin* create ventral-dorsal BMP gradient, wherein an intermediate amount of BMP signaling dictates the formation of presumptive neural plate border.	Hammerschmidt et al. (1996), Neave et al. (1997), Nguyen et al. (1998), Schumacher et al. (2011)
Neural Plate Border Induction	BMP, Wnt, and FGF signaling induce neural plate border specifiers (e.g., *pax3a*, *zic3*). BMP signaling induces *msx1a/1b/3* expression at neural plate border, which both induces NC specifier genes (*sox10*, *snai2*) and refines the neural/non-neural border by opposing *dlx* activity.	Tríbulo et al. (2003), Phillips et al. (2006), Garnett et al. (2012)
Neural Crest Formation	*msx1a/1b/3* are required for migratory neural crest survival. *tgfb3* is required for neural crest survival. *id3* is expressed in pre- and post-migratory neural crest. BMP signaling, via id2a, restricts ectomesenchyme identity in pre-migratory neural crest by inhibiting the expression of twist1a/1b.	Dickmeis et al. (2002), Phillips et al. (2006), Das and Crump (2012)
Parhyngeal Endoderm Morphognesis	BMP signaling, via *bmp2b*, induces the formation of *nkx2.3*+ pharyngeal endoderm. During somitogenesis, BMP signaling makes pharyngeal endoderm competent to respond to FGF signaling, which influences pharyngeal arch endoderm morphogenesis.	Lovely et al. (2016), Li et al. (2019a)
Pharyngeal Arch Patterning	Wnt signaling induces BMP signaling in ventral pharyngeal arch. Early in development (18–20 hpf), BMP induces Endothelin signaling in ventral arches to induce ventral identity. Later (24 hpf onwards), BMP and Endothelin signaling occupy distinct domains, where BMP signaling induces ventral arch identity and Endothelin induces intermediate arch identity. *grem2* and BMP signaling reciprocally inhibit one another to maintain dorsal, intermediate, and ventral arch identities. The scaffolding protein Wdr68 promotes ventral BMP activity by inhibiting TGF-β.	Alexander et al. (2011), Alexander et al. (2014); Zuniga et al. (2011); Alvarado et al. (2016)
Neurocranium Formation	BMP is necessary for formation of trabeculae from 16 to 18 hpf, and ectopic BMP signaling results in ectopic trabeculae cartilage. BMP signaling is necessary for *gata3* expression in the maxillary domain, which, in turn, regulates trabeculae formation.	Alexander et al. (2011), Swartz et al. (2021)
Cartilage Morphogenesis	*msx1a*, *bmp2b*, *bmp4*, *tgfb2*, and *tgfb3* are expressed in spatial and temporal patterns consistent with regulating ethmoid plate and Meckel’s cartilage morphogenesis. Knockdown of *tgfb2* results in shortening of the ethmoid plate and Meckel’s cartilage. s*mad5* mutants almost completely lack an ethmoid plate and show severe shortening and clefting of Meckel’s cartilage.	Swartz et al. (2011)
Joint formation	*gdf5* is expressed in the presumptive jaw joint, but *gdf5* mutants do not have an obvious jaw joint phenotype. *gdf6* is expressed along the ventral midline of the zebrafish craniofacial skeleton, and the articulations of the ceratohyals with the basihyal and the ceratohyals with the hypobranchials are disrupted in gdf6a mutants.	Reed and Mortlock (2010), Waldmann et al. (2022)
Suture Biology	Transgenic zebrafish lines reveal that human variants associated with craniosynostosis lie in enhancers that drive *bmp2/bmper* expression in suture osteogenic fronts, implicating BMP signaling in zebrafish suture development. Additional zebrafish studies on suture homeostasis are emerging.	Laue et al. (2011), Teng et al. (2018), He et al. (2023)

### Early development

#### Neural crest induction and migration

**FIGURE 3:**
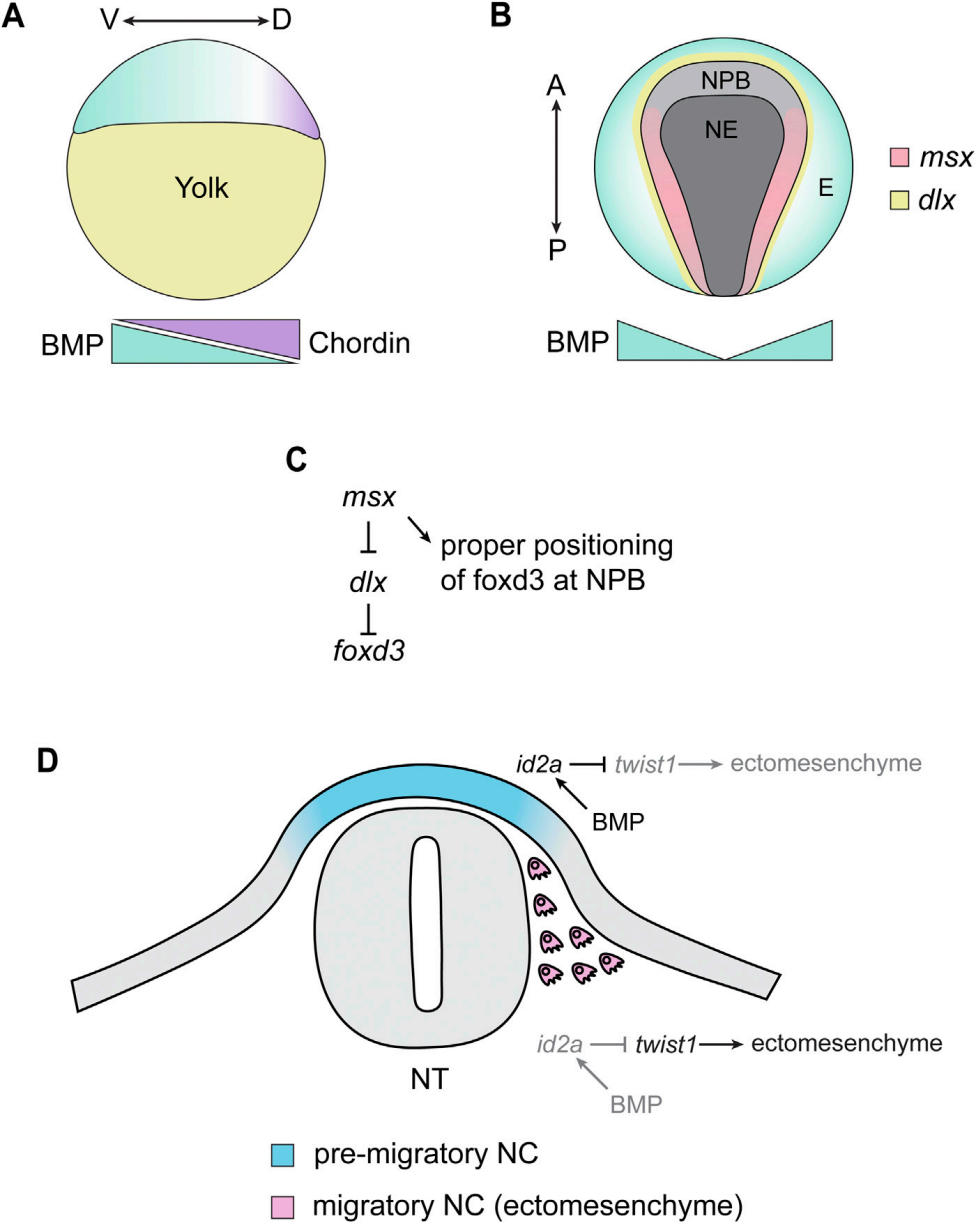
Summary of Zebrafish Studies on TGF-β Signaling and Neural Crest Induction/Migration. **(A)** During the late blastula stage (∼4 hpf), BMP ligands are expressed in the presumptive ventral side of the embryos (green). This is counteracted by chordin, which is expressed in the presumptive dorsal side of the embryo (purple). This creates a gradient of BMP signaling, where the presumptive ventral region of the embryo contains a high level of BMP signaling and the dorsal-most side of the embryo is absent of BMP signaling. V = ventral, D = dorsal. **(B)** The gradient of BMP signaling established at the blastula stage induces distinct tissue identities depending on the level of BMP signaling in that area, where the absence of BMP signaling allows for the development of neuroectoderm (NE), high BMP signaling induces epidermal (E) identity, and a moderate amount of BMP (together with Wnt and FGF signaling) induces the formation of the neural plate border (NPB). BMP signaling drives the expression of NPB transcription factors, including *msx1a/1b/3*, which are expressed within the NPB adjacent to the expression of *dlx* transcription factors. A = anterior, P = posterior. **(C)** In the NPB, *msx* transcription factors inhibit the activity of *dlx* transcription factors, which inhibit the expression of the neural crest specifier *foxd3.* This results in *foxd3* being expressed in the neural plate border, allowing neural crest induction to proceed. **(D)** During the migratory phase of neural crest development, BMP signaling restricts the ectomesenchyme potential of neural crest cell. In dorsal, pre-migratory neural crest (NC) cells, ectodermal BMP signaling induces the expression of *id2a* in the NC. This, in turn, inhibits the activity of *twist* genes, preventing the acquisition of ectomesenchymal identity. As NC cells delaminate and migrate away from this ectodermal BMP, *id2a* expression is lost in NC cells, allowing *twist* genes to activate an ectomesenchymal gene expression program in the NC. All images are oriented with anterior facing left and dorsal facing up unless otherwise specified.

Neural crest cells, the precursors of the craniofacial skeleton, arise from the neural plate border (NPB). As is the case in other vertebrates, the presence and position of the zebrafish NPB is established by gradients of inductive signals that pattern the ectoderm during gastrulation. Forward genetic screens performed in zebrafish produced several mutants that were necessary for establishing dorsal-ventral patterning in the early embryo (Hammerschmidt et al., 1996; Mullins et al., 1996). Subsequently, the genes mutated in these mutants were found to encode components of the BMP signaling pathway, including *swirl* (*bmp2b*), *snailhouse* (*bmp7a*), *somitabun* (*smad5*) and *dino* (*chordin/chd*) (Kishimoto et al., 1997; Schulte-Merker et al., 1997; Hild et al., 1999; Schmid et al., 2000). Like other vertebrates*,* a gradient of BMP signaling is established in the early zebrafish gastrula; BMP ligands are expressed in the ventral gastrula, whereas *chd* is expressed in the dorsal side, creating a gradient of high to low BMP signaling activity along the ventral to dorsal axis of the embryo (Hammerschmidt et al., 1996; Neave et al., 1997) (Figure 3A). This gradient results in an intermediate amount of BMP activity in the region adjacent to the neural plate, which is sufficient to form the neural plate border and, thus, the neural crest (Schumacher et al., 2011) (Figure 3B). The formation of the neural plate border is highly sensitive to the amount of BMP activity: an absence in BMP signaling results in a failure of NPB establishment, whereas a mild reduction results in the expansion of the NPB (Nguyen et al., 1998).

BMP signaling, together with Wnt and FGF, initiate the expression of transcription factors including *pax3a* and *zic3,* which confer NPB identity to the ectoderm (Garnett et al., 2012). BMP signaling seems particularly important for the expression of Muscle Segment Homeobox (*msx*) transcription factors. *msx1a, msx1b,* and *msx3* are all expressed at the neural plate border and are necessary for the expression of the NPB specifier *pax7* and the neural crest specifiers *snai2* and *sox10* (Tríbulo et al., 2003; Phillips et al., 2006) (Figure 3B). Although *msx1a/1b/3* are not necessary for the expression of *foxd3,* the domains of *foxd3* in the presumptive neural crest are closer to the midline in morphants compared to controls, suggesting that *msx* establishes and refines the neural-non neural border by opposing Distalless Homeobox (*dlx)* transcription factor activity (Phillips et al., 2006) (Figures 3B, C). *msx* gene expression is regulated by BMP signaling at the neural plate border; *msx1b* expression is eliminated in embryos that are BMP deficient, but in *swirl, snailhouse,* and *somitabun* mutants, where BMP signaling is present but reduced, the expression domain of *msx1b* is expanded, suggesting that *msx1b* is induced by a moderate amount of BMP and that the domain of *msx1b* is sensitive to gradients of BMP activation and inhibition (Tríbulo et al., 2003). In addition to their role in NPB patterning, *msx1a/1b/3* also appear to be necessary for survival and induction of neural crest cells well after NPB induction (14–20 hpf) (Phillips et al., 2006). This results in a reduction of migratory neural crest and a disruption in neural crest-derivatives, including the craniofacial skeleton (Phillips et al., 2006). Therefore, *msx* transcription factors have multiple roles in neural crest development that extend well past NPB induction.

Near the end of zebrafish gastrulation (∼11 hpf), the cells of the neural plate border are specified to become neural crest. There is strong evidence that TGF-β/BMP modulates pre-migratory neural crest cell specification. *tgfb3* is required for the survival of pre-migratory cranial neural crest cells in zebrafish, and knockdown or overexpression of *tgfb3* results in cell death and abnormalities in the craniofacial skeleton (Cheah et al., 2010). *id3,* a member of the Inhibitor of Differentiation (*id*) family of transcription factors, is expressed in the pre-migratory and post-migratory cranial neural crest cells (Dickmeis et al., 2002). Given that *id*s are frequently targets of BMP signaling, this likely represents direct inductive signaling by BMP on pre- and post-migratory neural crest cells. Furthermore, BMP has also been shown to be critical for restricting the ectomesenchyme identity of neural crest cells in the head (Das and Crump, 2012). *id2a* is expressed in the pre-migratory neural crest, consistent with a high level of BMP activity in this region (Das and Crump, 2012) (Figure 3D). *id2a* becomes excluded from the migratory neural crest, and this exclusion of *id2a* was shown to be necessary for *twist1a* and *twist1b* activity (Figure 3D) (Das and Crump, 2012). *twist1 and twist1b* are critical for the switch from ectodermal to ectomesenchyme identity in cranial neural crest cells, and knockdown of *twist1a/1b* results in fewer ectomesenchyme neural crest cells (Das and Crump, 2012). Consistent with this, knockdown of *twist1a/1b* or forced activity of BMP in migratory neural crest results in profound loss of many elements of the craniofacial skeleton (Das and Crump, 2012). Thus, BMP signaling plays a critical role in restricting ectomesenchyme identity in the pre-migratory neural crest, thereby ensuring that the switch from ectoderm to ectomesenchyme is properly regulated.

#### Pharyngeal endoderm formation and signaling

After specification, neural crest cells undergo an epithelial to mesenchymal transition (EMT) that allows them to detach from the dorsal neural tube and initiate migration. As migratory cranial neural crest cells arrive in the PAs, they receive inductive signals from surrounding tissues that provide positional information, allowing the neural crest to form morphologically distinct structures based on their position in each arch. This includes diffusible inductive signals from the surrounding PA tissues, which encompass the mesoderm, the ectoderm (which forms the pharyngeal clefts), and the endoderm (which forms the pharyngeal pouches) (Graham, 2003). Studies performed in zebrafish have shown that signals from pharyngeal endoderm are particularly important for patterning and morphogenesis of the craniofacial skeleton, and proper morphogenesis of the endoderm itself is necessary for these processes (Piotrowski and Nüsslein-Volhard, 2000; David et al., 2002). BMP signaling has been identified as an inductive signal necessary for proper morphogenesis of the pharyngeal endoderm (Lovely et al., 2016; Li et al., 2019b). *nkx2.3* is expressed in the prospective pharyngeal endoderm at the early somite stages and marks a subpopulation of the pharyngeal endoderm (Li et al., 2019b). Ablation of *nkx2.3*+ endoderm results in the loss or reduction of many cartilage elements in larval zebrafish, indicating that this subpopulation is necessary for craniofacial development (Li et al., 2019b). The specification of this subpopulation is dependent on BMP signaling; specifically, BMP signaling via *bmp2b* is necessary for the formation of these cells and proper pharyngeal endoderm development (Li et al., 2019b). In addition to its role in pharyngeal pouch specification, BMP signaling is also important for morphogenesis of the pharyngeal pouches during somitogenesis (Lovely et al., 2016). Blocking BMP signaling from 10 to 18 hpf results in a failure of pharyngeal pouch out-pocketing with no effect on endoderm formation, proliferation, or cell death, indicating that BMP signaling is necessary for the formation of the pharyngeal pouches (Lovely et al., 2016). Inhibition of BMP signaling during this period, in turn, influences craniofacial development, with many cartilage elements missing or reduced in BMP-deficient embryos (Lovely et al., 2016). BMP signaling is necessary for the expression of the FGF receptor *fgfr4* in the PAs, and inhibiting BMP signaling lowers the response to FGF in the pharyngeal endoderm, suggesting that BMP signaling makes PA endoderm cells competent to respond to FGF signaling (Lovely et al., 2016). Taken together, these studies indicate that BMP signaling is necessary for both the induction and morphogenesis of the pharyngeal endoderm, which, in turn, regulates the formation of the craniofacial skeleton.

#### Pharyngeal arch patterning

**FIGURE 4:**
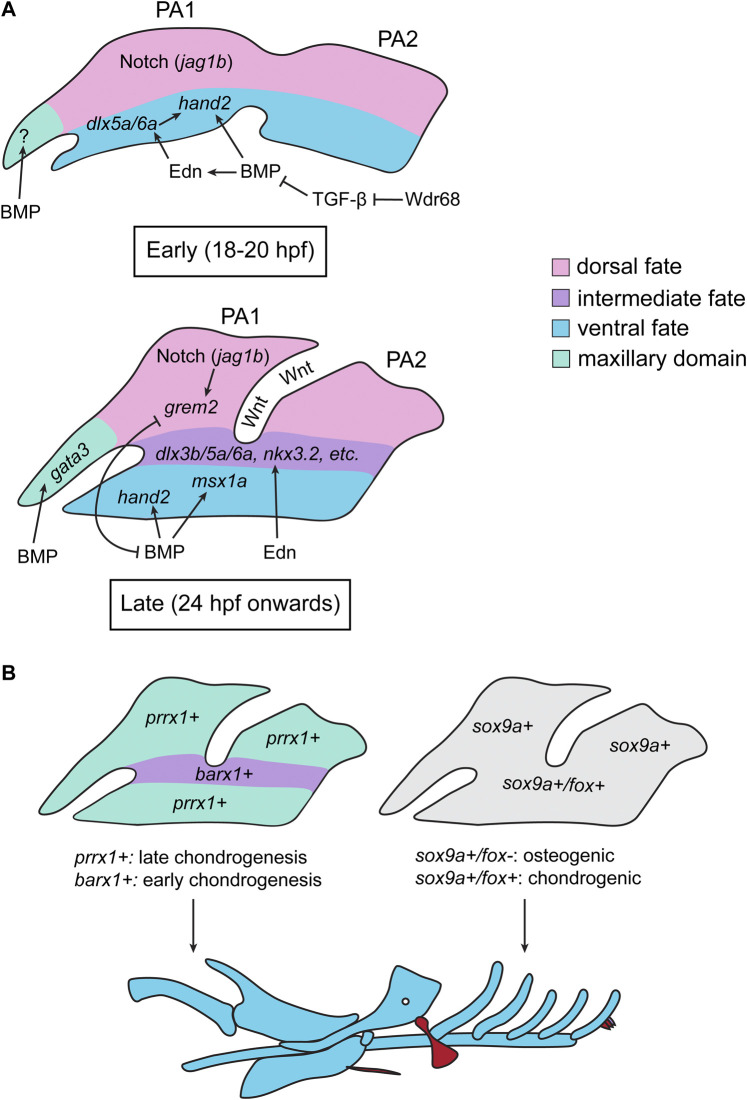
Summary of Zebrafish Studies on TGF-β Signaling and Pharyngeal Arch Patterning. **(A)** During the early stages of pharyngeal arch (PA) patterning (18–20 hpf), a ventral source of BMP activates Endothelin (Edn) signaling, which induces the expression of ventral-promoting transcription factors in the PAs (*dlx5a/6a, hand2*). BMP signaling also directly induced the expression of *hand2* in the ventral PAs. Notch signaling (via *jag1b*) specifies the dorsal domain. There is also evidence that BMP signaling regulates the formation of the trabeculae in the maxillary domain (M) during this period, however how this is achieved in zebrafish in unclear. BMP is opposed by TGF-β signaling during this period, and the scaffolding protein Wdr68 permits BMP signaling by sequestering Smad2/3 and thereby inhibiting TGF-β signaling. In later stages of PA patterning (24 hpf onwards), the BMP (induced by Wnt signling) and Edn pathways act independently: BMP signaling activates the expression of ventral (*hand2*) and intermediate-ventral (*msx1a*) genes, whereas Edn induces the expression of intermediate identity specifiers (*dlx3b/5a/6a*, *nkx3.2*). Dorsal Notch signaling induces the expression of *grem2*, which inhibits BMP signaling and restricts *hand2*/*msx1a* to the ventral-most region of the arches. In turn, BMP signaling restricts the expression of *grem2* to the dorsal region of each arch, creating distinct dorsal, intermediate, and ventral domains of gene expression. During this stage, BMP (together with Hedgehog signaling) also induces the expression of *gata3* in the maxillary domain **(B)** The gene expression domains generated in **(A)** result in differential domains of *prrx1* and *barx1* expression, with *barx1* being expressed in the intermediate domain and *prrx1* being expressed in the dorsal and ventral domains (left). This, in turn, results in heterochronic onsets of chondrogenesis, where *barx1+* cells initiate chondrogenesis earlier than *prrx1+* cells. This heterochrony, in part, dictates the shape/identity of the resulting skeletal elements. The spatial gene expression domains in the PAs also create differential regions of *fox* gene expression, which dictate whether a cell will undergo osteogenesis or chondrogenesis; *sox9a+/fox-*regions will initiate an osteogenic gene expression program, whereas *sox9a+/fox +* cells will initiate a chondrogenic program (right). All images are oriented with anterior facing left.

Although neural crest cells receive anterior-posterior positional information from cues that pattern the dorsal ectoderm (e.g., the Hox code), the dorsal-ventral pattern of the viscerocranium is acquired after migratory neural crest cells infiltrate the PAs (Minoux and Rijli, 2010). Studies in zebrafish and mouse have revealed that BMP, Endothelin, and Notch signaling in the PAs regulate the nested expression of *msx*, *hand*, and *dlx* transcription factors (Clouthier et al., 1998; Clouthier et al., 2000; Miller et al., 2000; Clouthier et al., 2003; Miller et al., 2003; Charité et al., 2001; Beverdam et al., 2002; Depew et al., 2002; Ozeki et al., 2004; Kimmel et al., 2007; Nair et al., 2007; Sato et al., 2008; Talbot et al., 2010; Zuniga et al., 2010; Alexander et al., 2011; Zuniga et al., 2011; Alvarado et al., 2016). This, in turn, confers ventral, intermediate, and dorsal identity to the PAs and determines the shape of the individual elements and the placement of important structures such as the jaw joint (Miller et al., 2003; Barske et al., 2016). In this model, BMP and Endothelin promote the development of the ventral and intermediate domains, whereas Notch signaling promotes the formation of the dorsal domain by excluding Edn1 and BMP signaling from the dorsal domains of the arches (Miller et al., 2000; Miller et al., 2003; Kimmel et al., 2007; Nair et al., 2007; Zuniga et al., 2010; Alexander et al., 2011; Zuniga et al., 2011; Alvarado et al., 2016) (Figure 4). Although mouse studies have clearly identified BMP signaling as an important regulator of PA patterning, the necessity of BMP signaling in axis patterning, NPB formation, and neural crest induction has precluded analysis of this signaling pathway at later stages (Dudas et al., 2004; Liu et al., 2004; 2005; Bonilla-Claudio et al., 2012). Zebrafish studies have allowed for the analysis of BMP signaling in this later process, as transgenic lines designed to monitor, overexpress, and deplete BMP activity have allowed researchers to circumvent the need for BMP in early development. BMP, induced, in part, by Wnt signaling, is active in post-migratory ventral PA neural crest cells (Alexander et al., 2011; Alexander et al., 2014) (Figure 4A). Zebrafish studies have revealed that BMP signaling in the PAs is biphasic (Alexander et al., 2011; Zuniga et al., 2011). During early arch development (∼18–20 hpf), BMP is necessary for *edn1* expression in the ventral arches, and depleting or ectopically activating BMP signaling during this time period reduces or increases *edn1* expression, respectively (Alexander et al., 2011) (Figure 4A). Together, Endothelin and BMP signaling synergistically induce the expression of ventral (*hand2*) and intermediate (*dlx5a/6a*) PA transcription factors, thus imparting ventral identity to the ventral PA neural crest cells (Alexander et al., 2011) (Figure 4A). In contrast, later in development (24 hpf onwards), *bmp4* and *edn1* are expressed in different domains, with *edn1* being slightly dorsal to *bmp4,* suggesting that these pathways pattern distinct domains at this timepoint (Zuniga et al., 2011). Consistent with this, ectopic activation of Endothelin or BMP signaling have distinct effects on the pharyngeal skeleton; while BMP activation induces the ventralization of many elements, Endothelin activation causes many elements to adopt an intermediate-like identity, indicating that BMP and Endothelin signaling promote ventral and intermediate identity, respectively (Zuniga et al., 2011). Accordingly, BMP promotes the induction of ventral-specifying factors (e.g., *msx1a* and *hand2*) while Endothelin signaling promotes the expression of intermediate domain-specifying factors, including signals required for positioning the jaw joint (e.g., *dlx3b, dlx5a, dlx6a*, and *nkx3.2*) (Alexander et al., 2011; Zuniga et al., 2011) (Figure 4A). Additionally, BMP signaling is no longer required for *edn1* expression past 24 hpf, further indicating that the two pathways pattern distinct domains later in PA patterning (Alexander et al., 2011; Zuniga et al., 2011). Dorsal Notch signaling, aided by intermediate-domain Endothelin signaling, drives the expression of *grem2* in the dorsal domain, thereby restricting BMP signaling to the ventral domain (Figure 4A) (Zuniga et al., 2011). BMP signaling, in turn, restricts *grem2* expression to the dorsal domain, thereby generating domains of high BMP in the ventral-most domain of the PAs and low BMP in the dorsal PAs, which reciprocally inhibit one another to maintain these domains (Figure 4A) (Zuniga et al., 2011). These PA domains, established by these signaling pathways and their interactions, shape the pharyngeal skeleton by promoting and restricting cartilage formation in distinct domains; Notch and BMP signaling promote the expression of *prrx1a/1b* and restrict the expression of *barx1* in the dorsal and ventral domains, whereas Endothelin signaling promotes the expression of *barx1* and inhibits the expression of *prrx1a/1b* in the intermediate domain (Barske et al., 2016) (Figure 4B). This creates a heterochronic onset of chondrogenesis in these domains–*barx1* in the intermediate domain will initiate chondrogenesis first, while *prrx1a/1* dorsal and ventral domains will prevent chondrogenesis and hold neural crest cells in a skeletal progenitor state (Barske et al., 2016) (Figure 4B). This heterochrony of cartilage induction is thought to, in part, shape the pharyngeal skeleton along the dorsal-ventral axis (Barske et al., 2016). Therefore, interactions between signaling pathways, including BMP, define distinct dorsal-ventral domains within the PAs which, in turn, determines the shape of each element based on its dorsal-ventral position in the PAs. Furthermore, several signaling pathways, including BMP, position the expression F-box (*fox*) transcription factors in PAs. This results in *sox9a+/fox+* domains in the PA, which promote a chondrogenic program, whereas dorsal *sox9a+/fox*- domains promote an osteogenic program that induce the formation of dermal bone (Xu et al., 2018) (Figure 4B). Therefore, BMP signaling, via the spatial deployment of *fox* genes, also patterns the PAs such that cartilage and bone are formed in distinct domains.

Unlike BMP signaling, which plays an undoubtedly critical role in establishing the early dorsal-ventral pattern of the pharyngeal skeleton, there is limited evidence in zebrafish that TGF-β or Nodal signaling mediates early PA development. However, a 2016 study found that the scaffolding protein Wdr68 is necessary for the induction of BMP signaling in the early ventral PAs and, therefore, plays a role in ventral arch identity (Alvarado et al., 2016). The authors showed that TGF-β signaling inhibits BMP signaling during early arch patterning, and that this inhibition was enhanced in *wdr68* mutants, suggesting that *wdr68* indirectly promotes BMP signaling by inhibiting TGF-β signaling in the ventral arches (Alvarado et al., 2016) (Figure 4A). Although it is unclear if *wdr68* modulates TGF-β signaling indirectly or directly, previous evidence has suggested that the Wdr68 protein binds and sequesters phosphorylated Smad2/3, indicating that this inhibition may occur at the level of Smads (Brown et al., 2008). Indeed, inhibitory interactions between BMP and TGF-β signaling is observed in other contexts, suggesting that this is a highly conserved mechanism of these two pathways (Reviewed by Hudnall et al. (2016)). It will be interesting to see if regulatory networks involving opposing functions of BMP and TGF-β signaling modulate other aspects of craniofacial and skeletal development.

#### Neurocranium formation

In addition to the pharyngeal skeleton, BMP signaling is also necessary for the early stages of neurocranium development, particularly the development of the trabeculae. Embryos lacking BMP signaling from 16 to 18 hpf have absent trabeculae, whereas ectopic BMP signaling results in malformation of the trabeculae and the formation of ectopic cartilage condensations on the trabeculae, indicating that BMP signaling likely induces the formation of trabeculae early in development of the neurocranium (Alexander et al., 2011). *gata3* mutants display a spectrum of phenotypes affecting the trabeculae, ranging from mild malformations in the trabeculae resulting from altered cell arrangements to complete absence of both trabeculae, similar to what is observed in BMP-deficient embryos (Alexander et al., 2011; Sheehan-Rooney et al., 2013; Swartz et al., 2021). *gata3* is expressed in the maxillary domain (the region that gives rise to the trabeculae) starting at 26 hpf, and *gata3* expression and function in the maxillary domain at this timepoint is dependent on BMP signaling, further indicating that BMP signaling mediates trabeculae formation via *gata3* (Swartz et al., 2021) (Figure 4A). However, attenuation of BMP signaling does not enhance the trabeculae phenotype in *gata3* morphants, suggesting that BMP signaling is not responsible for the phenotypic variability observed in *gata3* mutants (Swartz et al., 2021). Interestingly, treatment of *gata3* mutant embryos with SAG, an agonist of Hedgehog (Hh) signaling, ameliorates the trabeculae phenotypes, whereas treatment with cyclopamine (a Hh inhibitor) enhances the trabeculae phenotype (Swartz et al., 2021). Shh components were found to be upregulated in *gata3* mutants with a mild phenotype and downregulated in mutants with a severe phenotype, suggesting that Hh signaling, rather than BMP signaling, is a major contributor to the variability of phenotypes in *gata3* mutants (Swartz et al., 2021). This, in part, may explain the phenotypic variability observed in human craniofacial diseases, including those observed in individuals with mutations in the human paralog, *GATA3* (Swartz et al., 2021).

### Late development

#### Cartilage formation and morphogenesis

Once the initial pattern for the craniofacial skeleton is established and the primordia of each cartilage element has formed, the presumptive cartilage cells undergo cellular rearrangements, allowing them to fine-tune the shaping of the cartilage elements. This, in turn, creates distinct shapes for the cartilage elements that correlates to their function. For example, many elements, such as Meckel’s cartilage, the ceratohyal, and the trabeculae of the neurocranium adopt rod-like shapes, where chondrocytes adopt a stacked morphology, whereas the palatoquadrate, hyosymplectic, and ethmoid plate adopt fan-like shapes (Kimmel et al., 1998; Eames et al., 2013). In zebrafish, some of the most striking cellular rearrangements occur in the ethmoid plate. Neural crest cells that populate the frontonasal and maxillary processes will converge towards the midline and form the ethmoid plate and trabeculae. From 36 to 48 hpf, significant cell rearrangements occur within this population of neural crest cells, and by 48 hpf, the primordia of the ethmoid plate is visible (Dougherty et al., 2013). A study analyzing the expression of genes encoding signalling pathway components has revealed that members of the TGF-β superfamily are likely involved in this process in zebrafish. *msx1a,* a downstream target of BMP signaling, is expressed in the maxillary domain from 36 to 60 hpf, consistent with a role in morphogenesis of the anterior neurocranium (Swartz et al., 2011). Accordingly, *bmp2b* and *bmp4* are expressed in the anterior neural crest and oral ectoderm from 36 to 48 hpf (Swartz et al., 2011). Transcripts encoding the TGF-β ligands Tgfb2 and Tgfb3 are also expressed in regions and times relevant to ethmoid plate morphogenesis; *tgfb2* is expressed in the maxillary neural crest from 36 to 48 hpf, whereas *tgfb3* is expressed mainly in the oral epithelium surrounding the maxillary neural crest with only a small number of maxillary neural crest expressing *tgfb3* from 36 to 44 hpf. However, from 44 to 72 hpf, *tgfb3* is upregulated in the maxillary neural crest, suggesting that *tgfb3* has distinct early and late roles in ethmoid plate morphogenesis (Swartz et al., 2011). Taken together, the expression patterns of *tgfb2* and *tgfb3* in zebrafish are consistent with their roles in palatogenesis in mice (Kaartinen et al., 1995; Proetzel et al., 1995; Sanford et al., 1997). Consistent with their expression patterns, zebrafish deficient for these signaling pathways display craniofacial defects consistent with aberrant morphogenesis. While knockdown of *tgfb3* causes severe defects that preclude the analysis of the craniofacial skeleton, knockdown of *tgfb2* results in shortening of the anterior neurocranium, wherein the ethmoid plate and trabeculae are malformed (Swartz et al., 2011). Additionally, *smad5* mutant zebrafish, which have reduced BMP signaling activity, almost completely lack the ethmoid plate and trabeculae, which is consistent with mice lacking BMP signaling in palatogenic neural crest cells (Swartz et al., 2011; Kouskoura et al., 2013; Chen et al., 2019).

In addition to the ethmoid plate phenotypes observed in *tgfb2* morphants and *smad5* mutants, other cartilage elements also display aberrant morphology in these models. In *tgfb2* morphants, Meckel’s cartilage is severely reduced, which is consistent with its expression in mandibular neural crest cells (Swartz et al., 2011). *smad5* mutants have reduced Meckel’s and ceratohyal cartilages, with clefting occurring in the anterior of Meckel’s cartilage (Swartz et al., 2011). Therefore, it is likely that both TGF-β and BMP signaling play roles in shaping rod-shaped cartilage elements in addition to the trabeculae and ethmoid plate of the neurocranium (Swartz et al., 2011). However, how these pathways mediate cartilage morphogenesis in the zebrafish is currently uncharacterized. Previous studies in the zebrafish have indicated that two main processes fine-tune the shaping of cartilage elements during morphogenesis of the zebrafish craniofacial skeleton. The first is the acquisition of planar polarity, which mediates convergence-extension movements in the zebrafish craniofacial elements, allowing them to acquire their proper shapes. This is mediated by both Fat/Dchs signaling and noncanonical Wnt signaling, which are critical for the acquisition of chondrocyte polarity during morphogenesis of craniofacial cartilage (Kamel et al., 2013; Le Pabic et al., 2014; Sisson et al., 2015; Rochard et al., 2016; Ling et al., 2017; Dranow et al., 2023). The second is proper production and secretion of extracellular matrix proteins, which is intimately tied to chondrocyte identity. Mutations in *sox9a*, the zebrafish paralog of the cartilage master regulator *Sox9,* results in the formation of pre-cartilage condensations but prevents cartilage morphogenesis, indicating that cartilage identity is necessary for subsequent cartilage morphogenesis (Yan et al., 2002). Furthermore, mutations in components necessary for the synthesis or export of ECM components, particularly collagen, are necessary for cartilage morphogenesis, and aberration of these processes result in aberrant chondrocyte stacking and, thus, disrupted cartilage morphogenesis (Clément et al., 2008; Sarmah et al., 2010; Melville et al., 2011). One of the earliest identified biological outputs of TGF-β superfamily signaling is the regulation of ECM secretion, ECM modifying enzymes, and ECM interacting proteins (e.g., integrins) [Reviewed by Verrecchia and Mauviel (2002)]. Therefore, these pathways may regulate the timing of cartilage differentiation and/or cartilage ECM formation, thus regulating the shaping of these elements. Indeed, studies investigating the role of micro RNAs in zebrafish craniofacial development have provided interesting insights into the role of BMP signaling and cartilage morphogenesis: *mir92a* is necessary for the degradation of *nog3* mRNA, thus permitting the activation of BMP signaling in the PAs, which is necessary for the formation and morphogenesis of pharyngeal cartilages (Ning et al., 2013).

#### Joint development

Although zebrafish are emerging as a useful model for joint development and disease, studies in murine models have greatly informed our understanding of synovial joint development and have demonstrated a critical role for BMP and TGF-β signaling in this process. Prior to joint formation, the skeleton is laid down as uninterrupted primordia of mesenchymal skeletal progenitors. The first morphological sign of a developing joint is the formation of a condensation within these mesenchymal progenitors at the presumptive joint called the interzone (Pacifici et al., 2006; Koyama et al., 2008). As joint development proceeds, the interzone adopts a tri-layered structure, where the presumptive joint capsule is flanked by chondrogenic outer layers that give rise to articular cartilage (Pacifici et al., 2006; Koyama et al., 2008). Cells in the joint capsule are specified into progenitors for many joint structures, and the remaining joint capsule then cavitates to form the joint space (Pacifici et al., 2006; Koyama et al., 2008). During embryogenesis and post-natal life, joint progenitors mature into ligaments, tendons, menisci, the synovium, and articular cartilage, forming a mature joint (Pacifici et al., 2006; Koyama et al., 2008). The joint cavity also becomes filled with synovial fluid, which contains macromolecules (e.g., *Prg4*/Lubricin) that provide lubrication to the joint (Rhee et al., 2005; Koyama et al., 2014). Histological analysis of joints demonstrates a conserved tissue organization between mammalian joints and zebrafish craniofacial joints, and live imaging of joint development in zebrafish suggests that joint specification and cavitation occurs similarly in zebrafish and mammals, suggesting a homologous origin for teleost and mammalian joints (Askary et al., 2016). Accordingly, several joints in the zebrafish craniofacial skeleton express *prg4b* during the larval and juvenile periods, including the jaw joint, the hyoid joint, and the articulation between the ceratohyals and the basial, indicating a conservation of lubricated joints throughout evolution (Askary et al., 2016). Zebrafish lacking *prg4b* display a progressive loss of jaw joint integrity, suggesting that lubrication is necessary for the maintenance of zebrafish joints (Askary et al., 2016). Given the similarities between zebrafish and mammalian joint development and the rapid formation of zebrafish craniofacial joints, zebrafish are a potentially useful model for studying the mechanism of joint formation. However, caution should be used when extrapolating findings in zebrafish to mammals, as the zebrafish jaw joint is more similar to the incudomalleolar joint (the articulation between the incus and malleus in the mammalian middle ear) rather than the joints of the appendicular skeleton (Askary et al., 2016).

Arguably, development of the zebrafish jaw joint begins during early patterning of the first and second PAs. BMP signaling emanating from the ventral PAs is necessary for proper positioning of the jaw joint; *bmp4* overexpression or loss of *grem2b* abrogates *bapx1* expression in the intermediate domains of the PAs, which marks the nascent joint (Zuniga et al., 2011). Therefore, early events in arch patterning are directly responsible for the correct position of joints in the zebrafish craniofacial skeleton. Additionally, BMP signaling has been extensively linked to joint formation in murine models. In mouse, *Bmp2, Bmp4, Bmp7, Gdf5, Gdf6, Gdf7, Grem2,* and *Noggin* are all expressed in or around the joint interzone, with *Gdf5* being expressed in all joint interzones (Hogan, 1996; Wolfman et al., 1997; Brunet et al., 1998; Settle et al., 2003). Additionally, *Noggin* mutant mice show a widespread loss of joints throughout the body, indicating that inhibiting BMP signaling in the interzone is vital for joint formation (Brunet et al., 1998). This is consistent with zebrafish studies that show that overexpression of *bmp4* blocks the formation of joints in the craniofacial skeleton (Askary et al., 2015). Therefore, gradients of BMP activity must be precisely regulated to ensure the proper patterning and development of the nascent joint. A subgroup of BMP ligands, termed the GDF5/6/7 subgroup, appear to have particularly important (but complicated) roles in joint development. In mice, one of the first transcripts expressed in the interzone is *Gdf5,* and *Gdf5* is widely used as an early marker for the joint interzone (Storm et al., 1994). Interestingly, only a subset of joints are affected in mice mutant for *Gdf5* or *Gdf6,* and *Gdf5;Gdf6* double mutants do not phenocopy the widespread loss of joints observed in *Noggin* mutants, indicating that *Gdf5/6* are not necessary for the induction of all joints (Storm et al., 1994; Storm and Kingsley, 1996; Settle et al., 2003). Rather, several lines of evidence seem to suggest that *Gdf5/6/7,* particularly *Gdf5,* regulate BMP signaling in and around the joint interzone. It has been suggested that *Gdf5* competes with other BMPs for occupancy of *Bmpr1a* in the interzone but does not efficiently transduce a signal through this receptor, thereby preventing high BMP activity in the interzone (Lyons and Rosen, 2019). It has also been proposed that *Gdf5* induces BMP signaling through *Bmpr1b,* which is expressed in the articular cartilage but not the joint interzone (Lyons and Rosen, 2019). Additionally, *Gdf5* has been hypothesized to act as a “sink” for Noggin, preventing it from diffusing into the articular cartilage zone and thereby permitting BMP activity in this region (Lyons and Rosen, 2019). Investigations into *gdf5* and *gdf6a* (the zebrafish homologs of *Gdf5/GDF5* and *Gdf6/GDF6,* respectively) and their role in zebrafish skeletal and jaw development are emerging. In zebrafish, *gdf5* is expressed in the primary jaw joint, the ceratohyal, and in the future basihyal cartilage at 77 hpf, suggesting some role in skeletal patterning and joint formation in zebrafish (Reed and Mortlock, 2010). However, *gdf5* mutants do not display an overt craniofacial phenotype, suggesting either functional redundancy with other factors in these processes or a subtle phenotype that cannot be visualized with commonly employed histological methods (Waldmann et al., 2022). In contrast, *gdf6a* mutants do display a craniofacial phenotype; in *gdf6a* mutant zebrafish, the ceratohyal incorrectly articulates with the midline basihyal such that the ceratohyals appear offset rather than in-line with each other (Reed and Mortlock, 2010). Additionally, the hypobranchials are misshapen in *gdf6a* mutants, which results in lateral deviation of the ceratobranchials (Reed and Mortlock, 2010). In contrast to *gdf5,* which is expressed in laterally positioned joints in the craniofacial skeleton, *gdf6a* is expressed along the developing midline of the zebrafish craniofacial skeleton, suggesting potentially divergent roles for these signaling molecules relative to one another in zebrafish (Reed and Mortlock, 2010).

#### Calvaria development and suture homeostasis

Studies in murine models have implicated BMP signaling in regulating calvaria growth and suture homeostasis. Specifically, it is likely that BMP signaling plays a role in promoting osteogenesis in the suture. *Msx2,* a downstream target of BMP signaling, is expressed the osteogenic front of calvaria, and forced expression of both mutant and wild-type *Msx2* in the sutures results in premature suture ossification, indicating that inhibition of BMP signaling in the sutures is vital for the proper maintenance of suture mesenchyme (Liu et al., 1995). *Msx2* knockout mice display defects in the formation and ossification of calvaria, indicating that BMP signaling is necessary for ossification and must be regulated in the sutures to prevent premature fusion (Ishii et al., 2003; Han et al., 2007). Moreover, forced activation of BMP signaling in suture neural crest via expression of constitutively active *Bmpr1a* results in premature suture fusion, further suggesting that BMP signaling promotes ossification in the suture and must be precisely regulated to maintain suture homeostasis (Komatsu et al., 2013; Ueharu et al., 2023). Accordingly, the BMP antagonist *Noggin* is expressed in the suture mesenchyme, and misexpression of *Noggin* in the sutures or treatment of the sutures with recombinant Noggin protein delays suture closure (Warren et al., 2003; Shen et al., 2009). TGF-β also appears to be involved in this process; TGF-β, via Smad2/3, promotes suture closure and is regulated by Smad7 to prevent premature suture closure (Zhou et al., 2014). Deficiencies in other components of the BMP/TGF-β pathways (e.g., *Gdf6*) have been found to cause premature suture fusion, indicating that more components of the BMP/TGF-β pathways are likely involved in this complicated process (Settle et al., 2003). Although murine studies have been crucial for understanding the biology of cranial sutures and how suture closure is regulated, zebrafish are emerging as a model organism for understanding this process; zebrafish have been used to model Saethre-Chotzen syndrome (caused by mutations in *TWIST1* and *TCF12*) and craniosynostosis caused by alterations to retinoic acid synthesis (Laue et al., 2011; Teng et al., 2018). A recently published zebrafish study identified putative enhancers of *bmp2* and *bmper* (a regulator of BMP signaling) that drive reporter gene expression in the osteogenic fronts of transgenic zebrafish calvaria, suggesting that the function of BMP signaling in cranial development and suture homeostasis is conserved in zebrafish (He et al., 2023).

## Human craniofacial diseases and TGF-β signaling

As discussed in the previous section, BMP/TGF-β signaling plays a critical role in the specification, migration, patterning, and late cell behaviors of neural crest. Genetic analyses in humans strongly support such findings and demonstrate conclusively that craniofacial, skeletal, and joint diseases are caused by aberrant TGF-β superfamily signaling. Here, we review selected craniofacial, skeletal, and joint diseases and their association with BMP/TGF-β signaling. The information presented in this section is summarized in Table 3.

**TABLE 3:** Summary of Human Skeletal Conditions and their Associated Human Loci and Zebrafish Studies. Listed below are human craniofacial/skeletal diseases associated with aberrant TGF-β superfamily signaling, followed by their human gene symbol, their OMIM identifier, and references generated from human and zebrafish studies that implicate each gene/pathway in the associate disease. Refs. = References.

Disease	Gene	OMIM	Human Refs.	Zebrafish Refs.
Orofacial Clefting	*BMP4*	600625, 112262	Suzuki et al. (2009), Lumaka et al. (2012)	Alexander et al. (2011), Swartz et al. (2011), Swartz et al. (2021)
	*MSX1*	608874, 142983	Lidral et al. (1998), Jezewski et al. (2003), Suazo et al. (2004), Suzuki et al. (2004)	Phillips et al. (2006), Swartz et al. (2011)
	*NOG*	602991	Mangold et al. (2010)	Ning et al. (2013)
	*GREM1*	603054	Al Chawa et al. (2014)	NA
	*BMP7*	112267	Wyatt et al. (2010), Yu et al. (2015)	Alexander et al. (2011), Swartz et al. (2011), Swartz et al. (2021)
	*BMP2*	112261	Sahoo et al. (2011), Williams et al. (2012)	Alexander et al., 2011; Swartz et al., 2011; Swartz et al., 2021
Loeys-Dietz Syndrome	*TGFB2*	614816, 190220	Boileau et al. (2012), Gago-Díaz et al. (2014)	Swartz et al. (2011)
	*TGFB3*	615582, 190230	Rienhoff et al. (2013), Matyas et al. (2014), Bertoli-Avella et al. (2015)	Cheah et al. (2010), Swartz et al. (2011)
	*SMAD2*	619656, 601366	Zhang et al. (2017), Granadillo et al. (2018), Cannaerts et al. (2019)	NA
	*SMAD3*	613795, 603109	van de Laar et al. (2011), van de Laar et al. (2012)	NA
	*TGFBR1*	609192, 190181	Loeys et al. (2005), Loeys et al. (2006)	NA
	*TGFBR2*	610168, 190182	Loeys et al. (2005), Loeys et al. (2006), Drera et al. (2008)	NA
Myrhe Syndrome	*SMAD4*	139210, 600993	Caputo et al. (2012)	NA
Pierre Robin Sequence	*BMP2*	112261	Sahoo et al. (2011)	Alexander et al. (2011); Lovely et al. (2016)
	*BMP4*	112262	Capkova et al. (2017)	Alexander et al. (2011), Lovely et al. (2016)
	*BMPR1B*	603248	Yang et al. (2017)	Alexander et al. (2011), Alexander et al. (2014), Lovely et al. (2016)
Craniosynostosis	*MSX2*	604757, 123101	Jabs et al. (1993), Florisson et al. (2013)	NA
	*BMP2*	617439, 112261	Justice et al. (2012)	He et al. (2023)
	*BMP7*	112267	Justice et al. (2020)	NA
	*SMAD6*	617439, 602931	Timberlake et al. (2016), Timberlake et al. (2017), Timberlake et al. (2018); Calpena et al. (2020); Wu et al. (2020)	NA
Multiple Synostoses Syndrome	*NOG*	186500, 602991	Gong et al. (1999), Takahashi et al. (2001), van den Ende et al. (2005), Rudnik-Schöneborn et al. (2010)	Ning et al. (2013)
	*GDF5*	610017, 601146	Dawson et al. (2006)	Waldmann et al. (2022)
	*GDF6*	617898, 601147	Wang et al. (2016), Terhal et al. (2018)	Asai-Coakwell et al. (2007), Asai-Coakwell et al. (2009), Reed and Mortlock (2010)
Klippel-Feil Syndrome	*GDF3*	613702, 606522	Ye et al. (2010)	Ye et al. (2010)
	*GDF6*	118100, 601147	Tassabehji et al. (2008), Asai-Coakwell et al. (2009)	Asai-Coakwell et al. (2007), Asai-Coakwell et al. (2009); Reed and Mortlock (2010)
Osteoarthritis	*GDF5*	612400, 601146	Miyamoto et al. (2007), Valdes et al. (2011), Dodd et al. (2013), Styrkarsdottir et al. (2018), Tachmazidou et al. (2019)	Waldmann et al. (2022)

### Orofacial clefting and Pierre Robin Sequence

Orofacial clefting (OFC) is the most common craniofacial abnormality and one of the most common congenital abnormalities in the world with an incidence of 1.2/1000 live births worldwide (Martin and Swan, 2023). OFC is characterized by gaps in the primary palate (the lip, alveolus, and small region of hard palate anterior to incisive foramen) or the secondary palate (the remainder of the hard palate posterior to the incisive foramen and the soft palate) (Martin and Swan, 2023). OFC is a common feature of many syndromes; 1/3 of all OFC cases are associated with a syndrome (Martin and Swan, 2023). While there is some variability in the classification system used during diagnosis, OFC is commonly categorized by whether the clefting affects the lip, palate, or both, whether the clefting is bilateral or unilateral, and the degree to which the clefting affects the lip, palate, or both (Martin and Swan, 2023). OFC can also be categorized as syndromic (i.e., being associated with other phenotypes) or non-syndromic (i.e., presenting alone) (Martin and Swan, 2023). OFC of the primary palate results when the anterior maxillary processes fail to fuse with the nasal processes along the midline of the developing embryo, whereas OFC of the secondary palate occurs when the palatine shelves (the posterior aspect of the maxillary processes) fail to rotate and fuse at the midline (Martin and Swan, 2023). Palatal fusion involves the coordination of many biological processes including cell identity, migration, adhesion, death, and proliferation, and requires the careful coordination of gene expression and signaling pathway regulation (Martin and Swan, 2023; Won et al., 2023). Additionally, OFC usually occurs from a combination of environmental and genetic causes (Martin and Swan, 2023). BMP and TGF-β have been strongly implicated in the etiology of orofacial clefting in humans. Pathogenic mutations in *BMP4* have been associated with non-syndromic OFC, and microdeletions of 14q22-23, which encompasses *BMP4,* results in syndromic OFC (Suzuki et al., 2009; Lumaka et al., 2012). Moreover, mutations in *MSX1,* a downstream target of BMP signaling, have also been associated with OFC, further implicating this pathway in the etiology of OFC (Lidral et al., 1998; Jezewski et al., 2003; Suazo et al., 2004; Suzuki et al., 2004). Genome-wide association studies (GWAS) and burden analysis have implicated the BMP antagonists *NOG* and *GREM1* as genetic risk factors for non-syndromic OFC (Mangold et al., 2010; Al Chawa et al., 2014). *BMP7* mutations have also been associated with syndromic and non-syndromic OFC, and a deletion at 20p12.3 that encompasses *BMP2* has been linked to syndromic OFC (Wyatt et al., 2010; Sahoo et al., 2011; Williams et al., 2012; Yu et al., 2015). While there is limited evidence that variants in the TGF-β pathway cause non-syndromic OFC, skeletal features including OFC and other craniofacial abnormalities are occasionally found in patients with Loeys-Dietz syndrome, which are caused by mutations in *TGFB2, TGFB3, SMAD2, SMAD3, TGFBR1,* and *TGFBR2,* suggesting that this pathway is important for craniofacial development and disease (Loeys et al., 2005; Loeys et al., 2006). Finally, Myhre syndrome, which is partially characterized by the presence of OFC, is caused by mutations in *SMAD4,* implicating the TGF-β superfamily as a whole in OFC (Caputo et al., 2012).

Given the morphogenetic conservation between the zebrafish ethmoid plate and the mammalian hard palate, zebrafish has served as a useful model for studying the genetic and developmental origins of OFC (Swartz et al., 2011). BMP signaling plays multiple roles in ethmoid plate development, with alterations to BMP signaling in both early and late ethmoid development resulting in OFC-like phenotypes (Alexander et al., 2011; Swartz et al., 2011; Swartz et al., 2021). Perhaps the most useful role for zebrafish in studying palate formation and OFC is studying gene-environment interactions [Reviewed by Raterman et al. (2020)]. The ex-utero development of zebrafish facilitates manipulation of the developmental environment and observation of the subsequent effects on development. This also presents a unique opportunity to study gene-environment interactions; mutants with mild/no phenotype can be exposed to an environmental condition and observed to determine whether the OFC phenotype is worsened or ameliorated, allowing for the delineation of gene-environment interactions. Indeed, a screen to identify loci that are sensitive to alcohol exposure revealed that several genes interact with ethanol to produce a more severe craniofacial phenotype (Swartz et al., 2014).

Pierre Robin Sequence (PRS) is a craniofacial abnormality related to OFC (specifically cleft palate). PRS is characterized by mandibular hypoplasia (underdevelopment of the mandible), which results in a mispositioned tongue, leading to airway obstruction (Robin, 1923). PRS has an incidence of 1/8500 to 1/14000 live births and can severely impact the quality of life of affected individuals due to airway obstruction (Bush and Williams, 1983; Printzlau and Andersen, 2004). While the etiology of PRS is incompletely understood, it is hypothesized that PRS is caused, in part, by a failure of Meckel’s cartilage to elongate during embryogenesis, leading to a hypoplastic mandible. This prevents the tongue from being drawn forward during embryogenesis, resulting in a smaller oral volume (Seegmiller and Fraser, 1977). This reduced oral volume is predicted to prevent the palatal shelves from migrating close enough together at the midline, thereby preventing them from fusing and causing cleft palate (Seegmiller and Fraser, 1977). While genetic causes of PRS have not been completely elucidated, some patients with microdeletions at 20p12.3 and 14q22.1-q32.1, which encompass *BMP2* and *BMP4,* respectively, present with PRS, suggesting that BMP signaling plays a key role in mandible development and PRS (Sahoo et al., 2011; Capkova et al., 2017). Consistent with this model, patients with mutations in *BMPR1B* also present with PRS (Yang et al., 2017).

Studies in zebrafish suggest that aberrations to BMP signaling in several stages of craniofacial development may contribute to the development of PRS phenotypes and, therefore, OFC. Changes to neural crest induction can alter the number, migration, or survival of cranial neural crest cells can affect later craniofacial development, which can result in the reduction of Meckel’s cartilage and, therefore, a reduced jaw, suggesting that early events might contribute to the pathogenesis of this disease (Phillips et al., 2006; Cheah et al., 2010; Das and Crump, 2012). Additionally, formation of the pharyngeal endoderm, which requires BMP signaling, is necessary for the proper development of Meckel’s cartilage, suggesting that aberrant pharyngeal endoderm formation might explain the etiology of some cases of PRS (Lovely et al., 2016; Li et al., 2019b). Overactivation or elimination of BMP signaling during early PA patterning results in aberrant morphogenesis of several cartilage elements, including Meckel’s cartilage, implicating alterations to early PA patterning as a potential cause of PRS (Alexander et al., 2011; 2014; Zuniga et al., 2011). Knockdown and mutant studies have also demonstrated that both BMP and TGF-b signaling regulate fine-tuning of the shape of ventral elements, including the lower jaw. Determining how TGF-β and BMP signaling mediate this process will provide important insights into the pathogenesis of PRS (Swartz et al., 2011).

### Craniosynostosis

Craniosynostosis is another relatively common craniofacial abnormality associated with BMP and TGF-β signaling. Craniosynostosis affects 1/2100-1/2500 live births in the United States and is a common feature of many syndromes (Lajeunie et al., 1995; Boulet et al., 2008). Craniosynostosis occurs when the sutures (the fibrous joints that separate the flat bones of the skull) ossify prematurely, leading to reduced shock absorption and increased pressure on the growing brain (Johnson and Wilkie, 2011). This, in turn, can cause neurocognitive deficits or intellectual disability, as well as increased pressure on the sensory organs that are housed in the skull such as the eyes and ears, resulting in deficits in sensory organ function (Johnson and Wilkie, 2011). Craniosynostosis is typically characterized by the sutures that are fused in an individual, and the cause of craniosynostosis can be genetic, environmental, or a combination of both (Johnson and Wilkie, 2011). Multiple signaling pathways have been found to regulate suture fusion, and mutations in these signaling components frequently cause craniosynostosis in humans. There is mounting evidence to suggest that BMP signaling regulates suture maintenance and closure. Mutations in *MSX2* have been linked to Boston-type craniosynostosis (Jabs et al., 1993; Florisson et al., 2013). Additionally, haploinsufficiency of the BMP-specific inhibitory Smad *SMAD6* is predicted to be a modifier of the penetrance of craniosynostosis, with mutations in *SMAD6* increasing the risk of developing craniosynostosis (Timberlake et al., 2016; Timberlake et al., 2017; Timberlake et al., 2018; Calpena et al., 2020; Wu et al., 2020). GWAS has also identified *BMP2* and *BMP7* as susceptibility loci for craniosynostosis, further implicating this pathway as an important regulator of suture closure and craniosynostosis (Justice et al., 2012; Justice et al., 2020). Accordingly, whole exome sequencing of craniosynostosis patients reveals that potentially damaging mutations cluster in genes encoding components of the BMP signaling cascade (Timberlake et al., 2023). Although BMP signaling plays an undoubtedly important role in the etiology of craniosynostosis, many craniosynostosis cases are caused by mutations in components of the FGF signaling pathway (Johnson and Wilkie, 2011). Many of the variants in BMP signaling components (e.g., *SMAD6, BMP2, BMP7*) increase the risk of craniosynostosis rather than causing craniosynostosis directly, suggesting that interactions between pathways (namely, BMP and FGF) may contribute to the etiology of this disorder.

Studies have shown that zebrafish are a tractable model for studying suture biology and craniosynostosis, and the prospect of using zebrafish to study the suture biology and the pathogenesis of craniosynostosis is exciting (Laue et al., 2011; Teng et al., 2018). With respect to BMP signaling, a recent zebrafish study identified that GWAS variants associated with *BMP2* lie in putative enhancer regions for *BMP2* and *BMPER,* and that these enhancers likely drive the expression of these genes in the osteogenic fronts of the frontal bones, further implicating BMP signaling in suture homeostasis and craniosynostosis (Justice et al., 2012; He et al., 2023). Furthermore, yeast 2 hybrid assays indicated that the GWAS variants in these enhancers abrogate transcription factor binding ability, further suggesting that altered BMP signaling contributes to the pathogenesis of craniosynostosis (He et al., 2023). Moreover, unlike human sutures, which fuse by adulthood, zebrafish sutures remain patent throughout life; investigations into the mechanisms by which suture patency is maintained in zebrafish could inform the development of treatments or therapies for individuals affected by craniosynostosis and advance our understanding of stem cells in regenerative medicine (Topczewska et al., 2016). Indeed, recent studies in mouse have identified suture mesenchyme as a promising source of stem cells for regeneration of skeletal structures (particularly calvaria) and have demonstrated the role of BMP signaling in this process (Park et al., 2016; Vural et al., 2017). Complementing mouse studies with investigations into the mechanisms of zebrafish suture patency will surely advance our understanding of the etiology of craniosynostosis and provide insight into the use of suture stem cells as a therapeutic agent.

### Disorders of joints

While not strictly a craniofacial disease, diseases of joints are commonly comorbid with craniofacial abnormalities. For example, ossicle abnormalities are a common feature of multiple synostosis syndrome and some individuals with Klippel-Feil syndrome have laryngeal cartilage abnormalities. Additionally, the joints in the zebrafish craniofacial skeleton are increasingly proving to be a tractable system to study joint formation and disease.

#### Multiple synostoses syndrome

Multiple synostoses syndrome (MSS), also referred to as WL syndrome, deafness-symphalangism syndrome, or facio-audio-symphalangism syndrome, is a congenital skeletal disease characterized by multiple joint fusions, which frequently occur in the phalanges (Takahashi et al., 2001). Other common features of this syndrome are conductive hearing loss caused by fusion of the articulations between the ossicles and characteristic facial features, suggesting that this disease also affects components of the craniofacial skeleton (Takahashi et al., 2001). While the incidence of MSS is not known, it is predicted to be very rare (<1/1 000 000 live births) (OMIM #186500). The first identified causes of MSS were mutations in the BMP antagonist *NOG* (Gong et al., 1999; Takahashi et al., 2001; van den Ende et al., 2005; Rudnik-Schöneborn et al., 2010). Given that mutations in *NOG* also cause other skeletal diseases, including proximal symphalangism and brachydactyly, it has been proposed that *NOG* mutations cause a spectrum of diseases termed *NOG*-related symphalangism spectrum disorder (Potti et al., 2011). Additionally, mutations in the *NOG-*responsive ligands *GDF5* and *GDF6* also cause disease, but patients with mutations in *GDF5* lack the hearing loss observed in patients with mutations in *GDF6* and *NOG,* suggesting that *GDF5* does not regulate development of the ossicles (Dawson et al., 2006; Wang et al., 2016; Terhal et al., 2018)*.* MSS is predicted to be caused by haploinsufficiency of *NOG*, which results in an overactivation of BMP signaling in the growth plates and joint primordia, resulting in the overgrowth of the growth plates and fusion of the joints (Brunet et al., 1998). Accordingly, mutations in *GDF5* and *GDF6* that cause MSS frequently result in the overactivation of BMP signaling. For example, mutations in *GDF5* or *GDF6* that make their corresponding protein resistant to inhibition by NOG cause MSS, suggesting that the inhibition of BMP signaling via NOG is vital for joint development (Schwaerzer et al., 2012; Wang et al., 2016; Yu et al., 2023).

Joint development requires a fine balance of BMP signaling and for gradient strength and position to be regulated; both the loss of BMP ligands and antagonists of BMP signaling cause joint fusions, suggesting that a carefully orchestrated balance of BMP is necessary during joint development. Zebrafish are a system that is well-suited for the study of signaling gradients and morphogenesis. In zebrafish, several tools exist for studying the temporal and spatial dynamics of BMP signaling, including transgenic lines that allow for the observation of BMP signaling output (e.g., *Tg(BRE:GFP*)) or the temporal activation or attenuation of BMP signaling (e.g., *Tg(hsp70l:dnBmpr1a)* or *Tg(hsp70l:bmp4)*), allowing for the study of BMP signaling during the window of time that craniofacial jaw development is occurring (Alexander et al., 2011; 2014; Zuniga et al., 2011). Additionally, given its utility as a model for live imaging transgenic strains, zebrafish can be used to visualize all stages of joint formation in-real time, allowing for the delineation of the role of BMP signaling at each step of joint specification and morphogenesis and, as a result, providing insight into joint development and the etiology of diseases of joint fusion such as MSS. Indeed, studies have already taken advantage of the zebrafish model for studying joint development and have provided novel insight into the molecular mechanisms of chondrocyte maturation as it relates to joint development (Askary et al., 2015).

#### Klippel-Feil Syndrome

Klippel-Feil syndrome (KFS) is a rare congenital abnormality characterized mainly by the fusion of the cervical vertebrae, which causes a shortened neck, a limited range of motion in the neck, and a low posterior hairline (Klippel, 1912). Patients with KFS have also been reported to have laryngeal cartilage abnormalities, craniofacial abnormalities, and scoliosis (Clarke et al., 1994). While the reported incidence of KFS suggests it is rare (1/40,000 live births worldwide), many cases are not identified at birth and go unnoticed until an injury results in radiographic imaging of the neck, suggesting that the actual incidence of KFS is higher (Gruber et al., 2018). While mutations in the TGF-β superfamily ligands *GDF6* and *GDF3* have been shown to cause KFS, KFS can be genetically heterogeneous (Tassabehji et al., 2008; Asai-Coakwell et al., 2009; Ye et al., 2010). Historically, KFS was thought to be caused by defective cervical somite segmentation and differentiation into cartilage and bone, resulting in fused cervical vertebrae. However, while KFS is not explicitly a craniofacial abnormality, it has been suggested that the pathogenesis of KFS is neural crest-derived in nature; the part of the cervical spine that is commonly affected in patients with KFS is derived from the post-otic neural crest, and KFS can arise from abnormalities in neural crest fate choices in the cervical spine (Matsuoka et al., 2005). Zebrafish *gdf6a* mutants and *gdf3* morphants display phenotypes reminiscent of KFS, suggesting that zebrafish are a viable model for studying the role of these ligands in the etiology of KFS and their effect on post-otic neural crest activity (Asai-Coakwell et al., 2007; Asai-Coakwell et al., 2009; Ye et al., 2010). To date, Gdf3 and Gdf6 have yet to be studied regarding their potential roles in regulating of post-otic neural crest cell activity.

#### Osteoarthritis

Osteoarthritis (OA) is the most common joint disease worldwide, affecting up to 10% of men and 18% of women (Felson et al., 2000; Franke et al., 2023). OA is characterized by the breakdown of tissues in the joint, including articular cartilage, tendons and ligaments, the synovium (joint capsule), and bone, resulting in swelling of the joint, which leads to pain and reduced mobility (Brandt et al., 2009). OA is a major source of pain and disability worldwide, which, in turn, leads to increased socioeconomic burden (Woolf and Pfleger, 2003; Gupta et al., 2005; Schofield et al., 2013; Hunter et al., 2014). The main treatment courses for OA are pain management and surgical cartilage replacement, which, like any other invasive surgery, can result in complications (Nelson et al., 2014). OA is likely a complex disease, with the presence of multiple susceptibility alleles and environmental factors influencing the onset and severity of disease (Felson et al., 2000). Increased age and joint injury are the main risk factors for developing OA, but investigations into the causative factors of OA suggest that some individuals have a genetic predisposition to OA (Felson et al., 2000). Using several approaches, including GWAS, many loci have been identified as potentially conferring susceptibility to OA, including many members of the TGF-β superfamily. For example, GWAS have identified variants in the promoter of the key joint regulator *GDF5* as risk factors for OA (Miyamoto et al., 2007; Valdes et al., 2011; Dodd et al., 2013; Styrkarsdottir et al., 2018; Tachmazidou et al., 2019). Interestingly, many variants associated with OA risk are frequently found in non-coding regulatory elements rather than the open reading frames of coding genes, suggesting that slight alterations to the expression of genes, rather than the activity/function of the gene itself, are responsible for increased OA risk (Aubourg et al., 2022). Moreover, loss of *Grem1-*expressing chondrogenic progenitor cells in mouse joints results in an OA phenotype, further implicating altered BMP signaling in the progression of this disease (Ng et al., 2023). Additionally, it has been hypothesized that slight variations to the way the joint develops during embryogenesis (via modulating *GDF5* expression/activity) can influence how a joint functions in adulthood, which may influence the likelihood of developing OA later in life (Kiapour et al., 2018; Pregizer et al., 2018; Richard et al., 2020). Therefore, studying how joints develop during embryogenesis can provide insight into the risk factors for developing OA later in life. Additionally, many of the alterations in biological processes that lead to OA are developmentally relevant; understanding these developmental processes can increase our understanding of therapeutic strategies for OA.

While mammalian models have historically been used to study OA, zebrafish are gaining traction as a model for studying joint injury and disease. As stated previously, several of the joints in the zebrafish craniofacial skeleton are lubricated, and elimination of joint lubrication in zebrafish results in a progressive OA phenotype, demonstrating the tractability of zebrafish as a model for studying OA (Askary et al., 2016). The relatively short lifespan of zebrafish allows investigators to monitor joint health and integrity throughout their lifetime, allowing for relatively rapid analysis of joint maintenance under several conditions. Additionally, unlike mammals, zebrafish cartilage regenerates over the course of its lifetime; studying the molecular basis of this regeneration and injury repair can provide valuable insight into the development of preventative therapies and treatments for joint diseases like OA (Askary et al., 2016; Smeeton et al., 2022).

## Conclusions and future perspectives

Zebrafish have proven to be an invaluable resource for dissecting the molecular pathways that regulate craniofacial development and unraveling the pathogenesis of craniofacial abnormalities. They have also proved to be particularly valuable for investigating the role of critical signaling pathways, including BMP and TGF-β, in craniofacial development, due to the ability to manipulate signaling temporally to bypass the early necessity of these signaling pathways in gastrulation and body axis patterning. In recent years, researchers have harnessed the extrauterine development of zebrafish to create single-cell atlases of craniofacial development using single-cell RNA sequencing and to generate transgenic zebrafish lines to optogenetically control Nodal and BMP signaling (Fabian et al., 2022; Rogers et al., 2023). These resources will undoubtedly be paramount for furthering our understanding of signaling and craniofacial morphogenesis and complement other recent optogenetic and transcriptomic studies performed in different systems (Li et al., 2019a; Holmes et al., 2020; Farmer et al., 2021; Humphreys et al., 2023; Siewert et al., 2023; Sun et al., 2023). Zebrafish are also increasingly being used to model human diseases such as OFC, craniosynostosis, and OA, and may therefore provide valuable insight into the pathogenesis, treatment, and prevention of these diseases (Laue et al., 2011; Askary et al., 2016; Teng et al., 2018; Smeeton et al., 2022). Although studies in zebrafish and mouse have provided a wealth of information about the mechanisms underlying craniofacial development and disease, there are still unanswered questions about TGF-β/BMP signaling and their role in these processes. Much of what we know about the role of TGF-β/BMP in craniofacial development is based on the “canonical” Smad pathway. However, there is relatively little work, especially in zebrafish, examining the role of non-canonical TGF-β/BMP signaling in craniofacial development and disease. TGF-β/BMP ligands have been shown to activate TGF-β Activated Kinase (TAK1), which has been shown to regulate cellular behavior independent of transcriptional activation of target genes (Yamaguchi et al., 1995). Indeed, mice with TAK1-deficient cranial neural crest cells develop OFC through both Smad and non-Smad pathways (Song et al., 2013; Yumoto et al., 2013; Liu et al., 2018). Therefore, more investigation into the role of these noncanonical pathways in craniofacial development is warranted. Additionally, while many craniofacial diseases can be caused by mutations in components of a variety of different signaling pathways, the crosstalk between these pathways is frequently overlooked (e.g., the interaction between BMP and FGF in craniosynostosis). Investigating the interactions between multiple signaling pathways can inform both the etiology of disease in humans and the development of therapies or preventative measures that take signaling crosstalk into consideration.
